# Latrophilin-1-Mediated G_αq_ Signaling, Store-Operated Ca^2+^ Entry, and Ca_V_2.1 Activation Control Spontaneous Exocytosis at the Mouse Neuromuscular Junction

**DOI:** 10.3390/cells15090821

**Published:** 2026-04-30

**Authors:** Evelina Petitto, Frédéric A. Meunier, Sara Fidalgo, Cesare Colasante, Jennifer K. Blackburn, Richard R. Ribchester, Yuri A. Ushkaryov

**Affiliations:** 1 Medway School of Pharmacy, University of Kent and Greenwich, Central Avenue, Chatham Maritime ME4 4TB, UK; evelina.petitto@gmail.com (E.P.); jennifer.blackburn@yale.edu (J.K.B.); 2 Division of Cell and Molecular Biology, Imperial College London, Exhibition Road, London SW7 2AZ, UK; f.meunier@uq.edu.au (F.A.M.); saravsf@googlemail.com (S.F.); 3 Unité d’Embryologie Moléculaire, Institut Pasteur, Unités de Recherche Associées 2578, Centre National de la Recherche Scientifique, 25 Rue du Dr Roux, 75724 Paris, France; cesare@ula.ve; 4 Biomedical Sciences, University of Edinburgh, George Square, Edinburgh EH8 9JZ, UK; Richard.Ribchester@ed.ac.uk

**Keywords:** G-protein-coupled receptors, latrophilin-1, ADGRL1, neuromuscular junction, presynaptic, signaling, Ca^2+^ stores, Ca_V_2.1, exocytosis, neurotransmitter release, store-operated Ca^2+^ entry

## Abstract

**Highlights:**

**What are the main findings?**

- α-Latrotoxin mutant LTX^N4C^ triggers massive, burst-like increases in spontaneous exocytosis at motor nerve terminals by activating its G-protein-coupled receptor, latrophilin1.
- LTX^N4C^-induced exocytosis requires extracellular Ca^2+^ and relies on two signaling axes, subthreshold activation of G_aq_-mediated IP_3_ receptors and Ca_V_2.1-mediated Ca^2+^ influx, which together trigger Ca^2+^ from stores and subsequent store-operated Ca^2+^ entry.

**What are the implications of the main findings?**

- Massive spontaneous acetylcholine exocytosis can be triggered at neuromuscular junctions by cytosolic Ca^2+^ elevations driven by store-operated calcium entry.
- Latrophilin1 can act as a key regulator of spontaneous neurotransmitter release, suggesting a broader role for this receptor in synaptic physiology.

**Abstract:**

Latrophilin 1 (LPHN1/ADGRL1), an adhesion G-protein-coupled receptor (GPCR), is the principal receptor for α-latrotoxin (αLTX), a toxin that triggers massive neurotransmitter release. However, its endogenous signaling mechanism remains elusive. Here, we dissect the LPHN1 signaling pathway at the vertebrate neuromuscular junction, using the pore-deficient αLTX mutant LTX^N4C^ as a selective agonist. Combining electrophysiological recordings from LPHN1 knockout mice with pharmacological inhibitors, calcium imaging, and biochemical assays, we delineate the cascade from receptor activation to spontaneous quantal acetylcholine release. We demonstrate that LPHN1 is specifically localized to the presynaptic membrane and mediates LTX^N4C^-evoked release. Upon activation, LPHN1 engages the G_αq_–phospholipase C pathway to generate inositol 1,4,5-trisphosphate (IP_3_), triggering Ca^2+^ release from intracellular stores via IP_3_ receptors. This store depletion activates store-operated Ca^2+^ entry (SOCE), providing sustained Ca^2+^ required for LTX^N4C^-induced burst-like exocytosis. We uncover distinct roles for Ca_V_2.1 and Ca_V_1 channels in initiating and sustaining this response. These findings establish LPHN1 as a GPCR that harnesses intracellular stores and SOCE to drive spontaneous neurotransmission, revealing a novel signaling paradigm for adhesion GPCRs in presynaptic function.

## 1. Introduction

The precise control of neurotransmitter release is fundamental to synaptic communication. Among the host of proteins that orchestrate this process, LPHN1 (ADGRL1, according to the new nomenclature [1]) was first isolated as the target of αLTX, a potent toxin from black widow spider venom that causes massive secretion of neurotransmitters. The ability to mediate the toxin’s effect established LPHN1 as a presynaptic adhesion GPCR implicated in the regulation of exocytosis [2,3,4,5,6]. However, despite this long-standing association, the endogenous mechanism of LPHN1 action has remained poorly understood.

The structure of LPHN1 is consistent with its dual role in both cell adhesion and G protein signaling. It belongs to the adhesion class of GPCRs, characterized by a long, modular N-terminal domain responsible for extracellular interactions and a canonical GPCR domain containing seven transmembrane regions. Like most other adhesion GPCRs, LPHN1 undergoes autoproteolytic cleavage at a GPCR proteolysis site within its ectodomain. This cleavage generates two fragments: an N-terminal fragment (NTF) and a C-terminal fragment (CTF) which contains the transmembrane domains. While these fragments remain associated non-covalently at the cell surface, their separation is thought to be a key step in some signaling events [7,8]. This unique architecture allows LPHN1 to translate extracellular cues into intracellular signals.

For decades, our understanding of LPHN1 function has been advanced by studies using its exogenous ligand, αLTX. The native αLTX is a tetramer that not only binds to presynaptic receptors like LPHN1 but also inserts into the plasma membrane to form non-selective cation pores, profoundly disrupting synaptic function [9,10]. This dual action has historically made it difficult to isolate the specific signaling consequences of receptor activation from the direct effects of the toxin pore. The development of a mutant toxin, LTX^N4C^ [11], provided a critical tool to overcome this limitation. LTX^N4C^ is unable to tetramerize and therefore cannot form membrane pores, but it retains the ability to bind presynaptic receptors with high affinity [12]. This allows LTX^N4C^ to act as a pure pharmacological agonist, enabling the study of LPHN1 signaling in isolation.

Previous work using LTX^N4C^ has established that selective activation of LPHN1 triggers robust neurotransmitter release. LPHN1 is predominantly expressed in neurons [2,13], and participates in αLTX-induced release of glutamate and γ-aminobutyric acid [6,14]. At the mouse neuromuscular junction (NMJ), this is manifest as a dramatic increase in the frequency of spontaneous quantal acetylcholine (ACh) release, characterized by distinct bursts of exocytosis [15]. Despite these well-described physiological outcomes, the intracellular signaling cascade linking LPHN1 activation to this burst-like release has remained poorly defined.

Early studies hinted at the involvement of G proteins, showing that LPHN1 can functionally couple to G_αq_ and G_αo_ [5] and that αLTX stimulation leads to a rise in IP_3_ levels [2]. Furthermore, the phospholipase C (PLC) inhibitor U73122 was shown to block αLTX-evoked release in synaptosomes and hippocampal slice cultures [5,6], suggesting that the G_αq_-PLC-IP_3_ axis is a crucial downstream effector. This signaling cascade—G_αq_ activation of PLC, leading to IP_3_ production—points to a central role for the endoplasmic reticulum, which serves as an intracellular Ca^2+^ store. Indeed, depleting Ca^2+^ stores with thapsigargin (TG) blocked the LTX^N4C^ effect [6].

IP_3_ triggers Ca^2+^ release from endoplasmic reticulum stores via IP_3_ receptors (IP_3_Rs). Store depletion, in turn, can activate store-operated Ca^2+^ channels (SOCCs) in the plasma membrane, initiating a process known as SOCE [16]. SOCE provides a sustained, global Ca^2+^ signal [17] that can be further amplified by Ca^2+^-induced Ca^2+^ release (CICR) through ryanodine receptors (RyRs) [18]. While SOCE is well-characterized in non-excitable cells and neuronal cell bodies and dendrites [19], its role and mechanism in presynaptic terminals remain much less explored [20]. In neurons, the ER extends into axons and nerve terminals, where it could support presynaptic SOCE [21]. In addition to replenishing Ca^2+^ stores, particularly during sustained neuronal activity [22], presynaptic SOCE could regulate vesicle priming and spontaneous exocytosis [20,23,24,25].

In this study, we leverage the specificity of LTX^N4C^ and a combination of electrophysiological, genetic, fluorescence microscopy, biochemical, immunological, and pharmacological approaches to dissect the signaling pathway by which LPHN1 regulates spontaneous quantal ACh release at the vertebrate NMJ. Using LPHN1 knockout (KO) mice, LTX^N4C^ as a selective agonist, and a panel of inhibitors targeting key nodes in Ca^2+^ signaling, we delineate the complete molecular cascade from receptor activation to exocytosis. Our findings reveal that LPHN1 orchestrates burst-like ACh release by engaging the G_αq_-PLC-IP_3_ axis to mobilize intracellular Ca^2+^ stores, which in turn activates SOCE, with distinct contributions from voltage-gated Ca^2+^ channels (VGCCs) in initiating and sustaining this response.

## 2. Materials and Methods

### 2.1. Materials

All reagents were from Merck Life Science UK Limited (Gillingham, Dorset, UK), unless otherwise stated. UBO-QIC was from the Institute of Pharmaceutical Biology (University of Bonn, Bonn, Germany). SQ22536, pertussis toxin (PTX), cholera toxin (CTX), and xestospongin C were from Tocris Bioscience (Bristol, UK). Tetramethylrhodamine-conjugated α-bungarotoxin (αBuTX), αBuTX Alexa Fluor 546, Alexa Fluor 647 labeling kit, and Fluo-4 acetoxymethyl (AM) ester were from Thermo-Fisher Scientific (UK Life Technologies Limited, Paisley, UK). ω-Agatoxin IVA, ω-conotoxin GVIA, and ω-conotoxin MVIIC were from Alomone Labs (Jerusalem, Israel). Electron microscopy reagents (paraformaldehyde, osmium tetroxide, and LR White resin) were from Electron Microscopy Sciences (Hatfield, PA, USA); glutaraldehyde was from TAAB Laboratories (Aldermaston, UK).

A rabbit polyclonal antibody against LPHN1 (RL1) was previously described [26]. Here, it was affinity purified on an immobilized fusion protein consisting of glutathione-S-transferase (GST) conjugated with the NTF of LPHN1.

### 2.2. Ethics, Animals, and Tissues

The animal research reporting guidelines at https://arriveguidelines.org/arrive-guidelines (accessed on 12 January 2026) were consulted during the design of this study, which was approved by the University of Edinburgh College of Medicine and Veterinary Medicine Local Ethics Committee under PIL I672EE516 (3 June 2015), PIL 60/1017 (7 May 2015), and PPL 60/4569 (9 December 2013). The primary objectives were to: (i) measure responses of ex vivo innervated tissue (with or without LPHN1 expression) to LTX^N4C^ and various inhibitors of signal transduction using electrophysiological recordings (Section 2.3), optical imaging with physiological indicators (Section 2.5), and biochemical assays (Section 2.7, Section 2.10 and Section 2.13); and (ii) determine LPHN1 localization using fluorescence and electron microscopy (Section 2.14 and Section 2.15). A subset of experiments, which was designed to confirm LPHN1 localization, involved sciatic nerve axotomy in live animals as a regulated procedure (Section 2.12).

For the ex vivo tissue experiments, approved by the Medway School of Pharmacy Ethics Committee (MSOP-395, 12 October 2017), male mice, aged 21–28 days, of two strains were used: C57BL/6J (wild-type, WT) and AG148 (LPHN1 KO, created in the lab earlier). Both were maintained at Charles River (Charles Rivers Laboratories, Margate, UK) in identical conditions and had the same health status. Mice were swiftly euthanized by cervical dislocation, brains removed and processed as described below (Section 2.10 and Section 2.13); flexor digitorum brevis (FDB) muscles were rapidly dissected with their nerve supplies, immersed in Buffer A (Section 2.3) with constant oxygenation with humidified O_2_ and used as described in Section 2.3, Section 2.5 and Section 2.14. While electrophysiology experiments were performed on male mice to minimize variance associated with the estrus cycle, pilot experiments on female mice yielded equal results. Denervation studies included both male and female mice without sex stratification, as no sex-dependent differences were observed.

Adult frogs (*Rana temporaria*) were purchased from Blades Biologicals (Edenbridge, Kent, UK). Frogs were anesthetized in a 0.1% tricaine methane sulfonate solution and killed by double pithing. Brains were removed and processed as specified in Section 2.13; cutaneous pectoris muscles with their motor nerves were dissected and treated as described in Section 2.14 and Section 2.15. The primary outcome measure for this experimental design was a change (or lack thereof) in miniature endplate potential (MEPP) frequency, cytosolic Ca^2+^ (Ca^2+^_cyt_) fluorescence, protein bands on Western blots, or immunofluorescence images.

For denervation experiments, Wld^S^ mice (C57BlWldS) were used. Wld^S^ mice, obtained initially from Harlan-Olac (Launton, Oxfordshire, UK), showed no overt behavioral phenotype [27], and were maintained as a breeding colony in animal care facilities of the University of Edinburgh [28]. In some offspring (Wld^S−/−^), the chimeric Wld^S^ gene reverted to WT, restoring Wallerian degeneration. At the start of the experiment, the animals weighed ~24 g, and their health and genetic status were certified by respective breeding facilities. All animals were naive to treatment and had not been subjected to previous procedures.

### 2.3. Electrophysiology

Spontaneous presynaptic activity was evaluated by continuous postsynaptic intracellular recordings from muscle fibers in FDB muscle preparations, as described previously [8]. The muscles were incubated at room temperature in a physiological Buffer A (in mM: 137 NaCl, 5 KCl, 2 CaCl_2_, 1 MgCl_2_, 10 HEPES, 5.6 glucose) or a calcium-free Buffer B (in mM: 137 NaCl, 5 KCl, 0.2 EGTA, 1 MgCl_2_, 10 HEPES, 5.6 glucose), with constant oxygenation. Resting membrane potential (V_m_) of muscle fibers was typically −70 mV immediately after impalement. Although the recorded V_m_ occasionally deteriorated with time due to leakage around the sharp electrode, this deterioration was localized to the impalement site, did not depend on treatment, and did not affect MEPP frequency or burst characteristics. MEPP amplitudes were measured in cells with an apparent V_m_ between −70 and −40 mV only; the average amplitudes were compared within corresponding 5 mV ranges of V_m_ for control and test experiments, and normalized to –70 mV using an experimentally determined calibration curve.

To identify the signaling underlying the LTX^N4C^-induced increase in MEPP frequency (the number of unitary events per second, Hz, in consecutive, non-overlapping 1 s bins), pharmacological agents were added to preparations in the specified buffer 15–20 min or 5 h (for PTX and CTX [29]) before LTX^N4C^. Following LTX^N4C^ addition, recordings typically continued for 60 min; if the LTX^N4C^ effect did not develop within this timeframe, recordings were extended to 90 min. In reverse-order experiments, LTX^N4C^ was added first, and once its effect was fully established and recorded (typically within 30 min), respective compounds were added, and recording continued for another 60 min.

### 2.4. Chelation of Ca^2+^_cyt_

For loading nerve-muscle preparations with intracellular Ca^2+^ chelators, 50 mM stock solutions of BAPTA-AM or EGTA-AM (Thermo-Fisher Scientific, Oxford, UK) were prepared in anhydrous DMSO with sonication and heating to 50 °C. These stocks were diluted with Buffer B containing 0.2% Pluronic F-127 (Thermo-Fisher Scientific, Oxford, UK) to obtain 5 mM (10X) loading solutions, which were also sonicated. Electrophysiology recordings: Muscle preparations were incubated with 500 μM BAPTA-AM or EGTA-AM for 40 min, washed with Buffer B, and incubated for a further 30–40 min in Buffer B to allow for intracellular chelator de-esterification. This was followed by the addition of LTX^N4C^ and 2 mM extracellular Ca^2+^ (Ca^2+^_e_), while V_m_ recording continued during all these steps.

Fluorescence recordings: Preparations were preloaded for 3 h with Fluo-4 AM via a suction pipette, as described in Section 2.5. Subsequently, 500 μM BAPTA-AM was added to the bathing buffer, and loading of both the dye and chelator proceeded for 30 min. The tissue was then washed and incubated for 30 min in Buffer B to allow for BAPTA-AM de-esterification, followed by the addition of αBuTX Alexa Fluor 546, LTX^N4C^ and 2 mM Ca^2+^ and another 10 min incubation necessary for the LTX^N4C^ signal to develop. The fluorescence of these pre-stimulated preparations was then recorded under a fluorescent microscope, as described in Section 2.5.

### 2.5. Ca^2+^ Fluorescence Recordings

FDB nerve-muscle preparations were microdissected, with the end of the medial plantar nerve freed from surrounding connective tissue. The preparations were pinned to glass-bottomed dishes coated with Sylgard (Dow Silicones UK Ltd., Barry, Wales, UK), positioning the tissue over the central glass-bottomed window, and bathed in Buffer A (see Section 2.3). For loading with the calcium indicator Fluo-4, the nerve was drawn into a plastic suction pipette and secured in place with silicone grease. The Fluo-4-AM dye was first dissolved in 10 μL of anhydrous DMSO containing 2% Pluronic, then diluted to a final concentration of 200 μM with 100 μL of serum-free medium supplemented with 0.2% Triton X100. This solution was introduced into the shaft of the pipette using a capillary. Preparations were incubated in the dark at room temperature for 3–4 h with surface oxygenation by a stream of humidified O_2_. To label synaptic sites, αBuTX Alexa Fluor 546 was added 15 min before the preparations were used. Following incubation, preparations were washed with Buffer A and transferred to an inverted fluorescence microscope (Nikon SE200; Nikon UK, Surbiton, Surrey, UK) equipped with a pco.pixelfly digital camera (Excelitas Technologies Corp., Cambridge, UK) and a multi-LED illumination system (OptoLED, Cairn Research Ltd., Faversham, Kent, UK) controlled by WinFluor V4.2.2 (University of Strathclyde, Glasgow, UK). Fluo-4 fluorescence was excited at 470/40 nm and detected using a 505 nm dichroic mirror and a 530/30 nm emission filter. αBuTX Alexa Fluor 546 was visualized with excitation at 565/50 nm, a 540/30 nm excitation filter, a 555 nm dichroic mirror, and a 580/40 nm emission filter. Preparations were viewed through a Nikon S Plan Fluor 40×/0.60 objective, and regions containing groups of NMJs and adjacent unstained muscle fibers were selected for imaging. Two-dimensional images of these regions were captured every 1 or 2 s. Recording began 15 min after the addition of 0.25 or 1 nM LTX^N4C^ in Buffer A (see Section 2.3). Fluorescence traces reflecting cytosolic Ca^2+^ levels were obtained from selected regions of interest (ROIs) in the time-lapse images using WinFluor. Fluorescence intensity was normalized to baseline values and expressed as ΔF/F_0_ in relative fluorescence units.

### 2.6. ScFv Antibody Interaction with LPHN1

Solid-phase binding assays. Recombinant soluble V5-tagged NTF of LPHN1 was stably expressed in mouse neuroblastoma N2a cells and secreted into the serum-free medium, which was collected. The 96-well Maxisorp Nunc^TM^ immunoplates (NUNC LIMITED Newport Pagnell, Buckinghamshire, UK) were precoated with 400 ng mouse anti-V5 monoclonal antibody (mAb) at 4 °C overnight, then blocked with 1% BSA in phosphate-buffered saline (PBS) for 1 h at room temperature, washed, and used to capture V5-NTF from the expression medium via the V5 epitope by one of the methods below. This solid adsorbent was then employed to assess binding interactions with αLTX and the single-chain variable fragment (scFv) antibody A1-myc.

Binding assay. The anti-V5-coated multi-well plates were incubated with 50 ng V5-NTF for 2 h in a serum-free medium, at room temperature. The plates were washed with PBS containing 0.05% Tween-20, then incubated for 1 h with serial dilutions of 10 μg/mL A1-myc, washed, and counterstained for 16 h at 4 °C using horseradish peroxidase (HRP)-conjugated anti-His antibody. Color signal was developed using a chromogenic HRP substrate, and the absorbance was measured at 450 nm. Controls included wells lacking without LPHN1-NTF or primary antibody.

Competition assays. To determine whether LTX competes with A1 for binding to LPHN1, two approaches were used. (i) Serial dilutions of V5-NTF (containing 50, 25, 12.5, 6.25, and 3.12 ng) in serum-free medium were incubated with the anti-V5-precoated plates as above. After washing, the plates were incubated for 20 min with αLTX (6.5 μg/mL in PBS) or BSA (6.5 μg/mL in PBS) as a negative control. (ii) A fixed amount of V5-tagged LPHN1-NTF (50 ng) was captured on the anti-V5-coated plates. Following washing, the captured protein was incubated for 20 min with increasing amounts of αLTX (31, 62.5, 125, 250, 500, and 1000 ng) in PBS or with equivalent amounts of BSA as a negative control. Finally, the plates prepared by method (i) or (ii) were washed and incubated for 1 h with 10 μg/mL A1-myc. Bound A1 was detected using HRP-conjugated anti-His antibody and quantified as above. The experiment was repeated 5 times, *n* = 5.

Data analysis. For the competition assay, binding curves were fitted to the data using the following Equation (1) (derived from [30]):

(1) $$f=\;\frac{B_{max}\times \left[\alpha LTX\right]}{K_{d}+\left[\alpha LTX\right]}$$

where *f* is the bound A1-myc signal, *B*_max_ is the maximum specific binding, and *K*_d_ is the apparent dissociation constant for αLTX binding. Curve fitting to determine *K*_d_ and *IC*_50_ was performed using SigmaPlot 15.0.013 (SSPS Inc., Clarion, PA, USA) and Prizm 8 (GraphPad Software, Inc., Boston, MA, USA).

### 2.7. Precipitation of Neuronal LPHN1 with A1-V5 Antibody

Cerebrocortical synaptosomes were prepared from rat brain as previously described [31]. The synaptosomes (P2 fraction) were solubilized on ice for 1 h in PBS containing 1% Triton X-100, protease inhibitors cocktail (Merck Life Science UK Limited), and 2 mM EDTA. The lysate was diluted 3-fold with PBS and incubated with 10 μg of A1-V5 antibody or buffer for 2 h at 4 °C with agitation. Subsequently, 100 μL of an anti-V5 agarose affinity gel (Merck Life Science UK Limited) was added, and incubation continued for a further 2 h. The samples were centrifuged, unbound material collected, and the samples washed with 0.3% Triton X-100 in PBS. The bound material was eluted with a 2X loading buffer for SDS-electrophoresis. Samples from all fractions were analyzed by SDS-electrophoresis and Western blotting.

### 2.8. Lack of A1 Antibody Interaction with αLTX

To verify that in the binding experiments above αLTX competed with A1 for the NTF of LPHN1 rather than binding to A1 itself, solutions of the recombinant V5-NTF or A1-V5 in a serum-free medium were incubated with 50 μL aliquots of an αLTX agarose affinity gel (Section 2.13) for 30 min, then washed by centrifugation and eluted with a 2× loading buffer for SDS-electrophoresis. The eluates were analyzed by Western blotting using an anti-V5 mAb and an HRP-conjugated anti-mouse IgG.

### 2.9. Sucrose Density Gradient Centrifugation

Step gradients of sucrose density (1–12%, 1–6% or 2–10%) were prepared in 50 mM Tris-HCl, 150 mM NaCl, 5 mM EDTA and 0.01% sodium azide (NaN3), pH 7.4, following the previously described method [26]. The gradients were formed by placing a “cushion” of 25% sucrose to the bottom of a centrifuge tube, followed by careful layering of 1 mL aliquots of progressively diluted sucrose solutions. Samples containing the proteins of interest were layered on the gradients and centrifuged in an SW 41 rotor centrifuge (Beckman Coulter, High Wycombe, Buckinghamshire, UK) at ~120,000× *g* for 20 h at 4 °C, or in a SW 55 rotor centrifuge (Beckman Coulter) at ~240,000× *g* for 14 h at 4 °C. The gradients were fractionated into 0.5 mL aliquots using a capillary, a peristaltic pump, and a fraction collector (Bio-Rad Laboratories). Aliquots from each fraction were analyzed by SDS-electrophoresis and Western blotting using anti-V5 mAb (at 0.2 μg/mL), and visualized using HRP-conjugated anti-mouse IgG (at 0.4 μg/mL).

### 2.10. RNA Extraction

Total RNA was isolated from mouse brain, spinal cord ventral horn (SCVH), and FDB muscles, as described previously [32]. (i) Brains were removed and homogenized using a Potter-Elvehjem homogenizer (Sartorius, Epsom, UK) in lysis buffer (E.Z.N.A. Total RNA Kit 1, Omega Bio-tek, Avantor, Inc., Lutterworth, UK). (ii) For spinal cord samples, the sacral segments of spinal cords, which innervate hind legs, were isolated and cut into 1 mm-thick cross-sections, as described previously [32]. The SCVHs were microdissected from each section under a binocular microscope, taking care to exclude white matter, transferred directly to lysis buffer, and homogenized as the brains. (iii) For muscle samples, the distal hindlimb muscles (including FDB) were removed from the bones and homogenized in lysis buffer using a rotor-stator homogenizer (Ultra-Turrax T25, Janke & Kunkel IKA-Labortechnik, Staufen im Breisgau, Germany).

All tissue homogenates were then processed for total RNA isolation using the E.Z.N.A. Total RNA Kit 1 and treated with DNase to remove genomic DNA contamination. RNA concentration and purity were assessed spectrophotometrically.

### 2.11. Quantification of mRNA Expression

First-strand cDNA synthesis and Reverse transcription–Quantitative PCR (RT-qPCR) were performed and verified as described previously [32]. Raw fluorescence data were analyzed using LinRegPCR version 20210614 software [33]. The initial amounts of target cDNAs in tissue samples were determined as described [32].

### 2.12. Denervation Experiments

Sciatic nerve section was carried out as previously described [28]. To denervate the hind foot muscles, mice were anaesthetized by halothane inhalation (2% in 1:1N_2_O/O_2_), the sciatic nerve was exposed and a 1–2 mm section was removed. Wounds were closed and secured with 6/0 silk sutures, and the mice were returned to their cages for recovery from anesthesia. After 48 h, mice were swiftly euthanized, in accordance with the UK Home Office regulations, Schedule 1. The rationale for this endpoint was that Wallerian degeneration normally eliminates motor terminals 48 h after the axotomy. All the distal hind leg muscles, including FDB, were quickly dissected and frozen until further use as specified in Section 2.13. The primary outcome measure under this experimental design was a change in LPHN1 band intensity on Western blots.

### 2.13. Western Blotting

To analyze the distribution of LPHN1–3 and neurexin Iα (NRXN1α) between the neuronal and muscle cells, brain and muscle tissues from WT and LPHN1 KO mice, or frog brains, were solubilized in 10 mM Tris buffer pH 7.4, containing 1% Thesit protease inhibitor cocktail and 2 mM EGTA. To aid solubilization, the tissues were homogenized in the solubilization buffer using a Potter-Elvehjem homogenizer (Sartorius, Epsom, UK) (brains) or a rotor-stator laboratory homogenizer (Ultra-Turrax T25, Janke & Kunkel IKA-Labortechnik) (muscles). All samples were centrifuged to remove un-solubilized residues and the supernatants analyzed by Western blotting directly or after enrichment. To concentrate the αLTX binding proteins, the lysates were incubated with 50 μL aliquots of an αLTX agarose affinity gel produced in the laboratory by attaching 1 mg αLTX to 1 mL of CNBr-activated agarose [26]. The gel was washed by centrifugation and eluted with 2X loading buffer for SDS-electrophoresis.

Western blotting was performed as described previously [7]. Briefly, the samples of solubilized tissues or eluates from affinity columns were analyzed by SDS electrophoresis in 8% polyacrylamide gels. The samples in 1X SDS-loading buffer were prepared by heating for 30 min at 50 °C. Electrophoretically separated proteins were transferred onto Immobilon^®^-P membrane using a wet electro-transfer unit (Bio-Rad Laboratories, London, UK). Target proteins were labeled with primary antibodies (anti-NTF antibody RL1, anti-CTF antibody R4 [34], or anti-V5 mAb), followed by HRP-conjugated anti-rabbit goat IgG or anti-mouse goat IgG (Sigma-Aldrich, Gillingham, Dorset, UK) secondary antibodies. The bands were visualized using a chemiluminescent substrate (Millipore, Merck Life Science UK Limited) and captured by a LAS-3000 Fujifilm gel imager (Raytek Scientific Ltd., Sheffield, UK). Positive bands were quantified using ImageJ (version 1.45m; National Institutes of Health, Bethesda, MD, USA) [35], while representative images were included in the figures.

### 2.14. LPHN1 Localization by Fluorescence Microscopy

LPHN1 immunolocalization at the amphibian NMJ was performed on cutaneous pectoris nerve-muscle preparations from *Rana temporaria*. The cutaneous pectoris (CP) muscles with their motor nerves were stretched, stretched to their resting length, pinned in a Rhodorsil-lined (Rhone-Poulenc, Reading, Berkshire, UK) plastic chamber and bathed in Ca^2+^-free Ringer solution (115 mM NaCl; 2.1 mM KCl; 1.8 mM MgCl2; 2 mM EGTA; 5 mM HEPES; pH 7.25) for 90 min. Individual muscle fibers were teased apart in PBS, incubated for 20 min in PBS supplemented with 10% normal goat serum and 0.2% Triton X-100, followed by incubation with the affinity purified anti-LPHN1 antibody RL1 in the same buffer for 16 h, at 4 °C. After extensive washing, the samples were counterstained for 1 h with secondary, FITC-conjugated anti-rabbit IgG (Sigma-Aldrich Company Ltd., Gillingham, Dorset, UK) and rhodamine-conjugated αBuTX to stain ACh receptors. Samples were then mounted with Fluoprep (BioMerieux UK Limited, Basingstoke, UK) and analyzed using confocal laser microscopy.

For axon removal experiments [36], connective tissue in amphibian muscle preparations was loosened by a 1 h treatment with 1 mg/mL clostridial collagenase I (Roche Products Limited, Welwyn Garden City, UK) in Ringer solution supplemented with 5 mM Ca^2+^, but devoid of Mg^2+^. During the incubation, the solution was periodically mixed by gentle pipetting. After this extended collagenase treatment, the nerve terminal was carefully lifted off the muscle fiber using a micropipette, physically removing the axon and exposing the underlying postsynaptic membrane. Preparations were then labeled for fluorescence microscopy as described above. Treatment with collagenase did not affect the LPHN1 labeling in preparations where the nerve was not removed.

Mouse NMJ labeling was carried out using three different approaches: (i) immunostaining of permeabilized FDB preparations with the anti-LPHN1 antibody RL1 overnight, followed by a wash and incubation with a goat anti-rabbit IgG labelled with Alexa Fluor 488; (ii) a 20 min binding of fluorescent LTX^N4C^ produced using an Alexa 647 labelling kit (Thermo-Fisher Scientific—UK, Life Technologies Limited, Paisley, UK), followed by an extensive wash; and (iii) staining of synaptic vesicle by a 5 min stimulation with Buffer A (adjusted to contain 55 mM KCl and 87 mM NaCl) in the presence of 10 μM FM1-43 dye (Thermo-Fisher Scientific—UK, Life Technologies Limited, Paisley, UK) [37,38]. In both cases, the ACh receptors were labeled by a 20 min incubation with αBuTX Alexa Fluor 546.

Images were captured under an upright microscope AxioSkop (ZEISS UK, Cambridge, UK) equipped with an LSM510 laser-scanning module (ZEISS UK), using the following configurations. Frog NMJ staining: Neofluar 20x/0.5 objective; excitation, 488 and 543 nm; emission, 515–565 nm and >560 nm. Mouse NMJ staining: Achroplan 40x/0.75 W Ph2 water-dipping objective; (i) for LPHN1 immunostaining: excitation, 488 and 543 nm; emission, 505–530 nm and >560 nm; (ii) for LTX^N4C^ Alexa Fluor 647 binding detection: excitation, 633 nm, emission, >670 nm; (iii) for FM1-43 detection in synaptic vesicles: excitation, 488 nm; emission, 505–530 nm; and (iv) for αBuTX Alexa Fluor 546 AChR staining: excitation 543 nm; emission, 560–615 nm or >605 nm filter.

### 2.15. Electron Microscopy

The full experimental protocol has been described previously [39]. In brief, frog cutaneous pectoris muscles were dissected as outlined in Section 2.2 and fixed with 4% paraformaldehyde. Specimens were then postfixed sequentially with (i) 4% paraformaldehyde, 0.1% glutaraldehyde, and 0.2% picric acid; and (ii) 0.5% OsO_4_. Tissues were dehydrated and embedded in LR White resin. Thin sections were reduced with 0.1% NaBH_4_, rinsed, and blocked with 5% BSA and 0.1% gelatin. Subsequently, specimens underwent the following steps: incubation with an affinity-purified rabbit anti-LPHN1 antibody for 120 min, counterstaining with goat anti-rabbit IgG conjugated to 5-nm colloidal gold particles for 90 min, wash, fixation with 2.5% glutaraldehyde, and post-fixation with 1% OsO_4_. Grids were counterstained with 1% lead citrate and 2.5% uranyl acetate and examined under a JEM-1010 electron microscope (JEOL (UK) Ltd., Welwyn Garden City, Hertfordshire, UK). Electron micrographs were digitized using an AGFA Duoscan T1200 scanner (AGFA UK, Uxbridge, Middlesex, UK).

### 2.16. Data Collection and Analysis

Electrophysiology: To minimize animal use and account for shipping and cohort-associated confounding factors, both FDB muscles from each animal were used in a within-animal paired design (one treated, one control), providing rigorous control for biological variability within each experimental day. The assignment of left or right muscle to treatment was randomized across animals to control for any potential lateral bias. To facilitate robust statistical analysis, muscles from 3–6 mice were used for each experimental condition (*n* = 3–6), and recordings from 5–10 muscle fibers were made during each phase of the experiment. With reference to sampling and statistical testing, *n* refers to the number of mice from which FDB nerve-muscle preparations were made (used for statistical calculations) and *N* refers to the number of muscle fibers sampled (used to determine the mean value for each mouse); both values are reported in the figure legends.

Sample sizes were determined a priori using G*Power 3.1.9.7 (Universität Kiel, Kiel, Germany). Because different experimental comparisons involved different effect sizes, separate power analyses were performed for each major outcome type. (i) For the primary electrophysiological comparison—bursts versus basal MEPP frequency—the effect size was ~100-fold, as reported previously [8]. A power analysis for a paired *t*-test (reflecting the within-animal design) indicated that 3 animals per condition would provide 80% power at α = 0.05 to detect this difference. (ii) Other comparisons involved smaller effect sizes, e.g., average MEPP frequencies, which included both bursts and inter-burst intervals (IBIs), exceeded average basal frequencies by 20–40-fold, while the effect size for intra-IBI frequencies was ~10-fold [8]. These experiments required 4–6 animals per condition to achieve 80–95% power, as indicated by a power analysis.

All other quantitative experiments were reproduced at least three times with three or more replicates (*n* = 3; *N* ≥ 9). Qualitative experiments (imaging and Western blotting) were repeated three or more times, with 3–9 individual NMJs or protein bands imaged per experiment and per condition.

The total numbers of animals used across different experiments were C57BL/6J, *n* = 220; AG148, *n* = 30; Wld^S^, *n* = 10; Wld^S−/−^, *n* = 4; frogs, *n* = 5. Specifically, the C57BL/6J mice were distributed across electrophysiology (*n* = 205, encompassing approximately 40–50 independent experimental conditions, with 3–6 mice per condition), calcium imaging (*n* = 9), and immunofluorescence (*n* = 6). To maximize data yield per animal, tissues for biochemical analyses (RT-qPCR, affinity chromatography and Western blotting) were harvested from the same animals whose muscles were used in electrophysiological experiments, and were frozen in liquid N_2_.

For the whole set of experiments, animals were sampled sequentially across multiple shipment dates and cohorts. This approach sampled variability across many unknown conditions and could enhance the general applicability (external validity) of all statistically significant findings. While this design could be less sensitive in detecting very small effect sizes compared to concurrent group randomization, given the large effect sizes observed in this study (typically >100-fold for primary comparisons) this trade-off was considered acceptable and the enhanced generalizability was prioritized.

In LTX^N4C^-only experiments, the investigators were aware of the stimulus identity during data collection, because its visible effects could not be concealed. However, in pharmacological tests, the experimenters were blind to the identity and concentration of the compounds applied. In addition, to mitigate bias, all datasets were analyzed independently by at least two analysts who were blinded to group allocation.

Statistical analysis was performed using GraphPad Prism version 8.0.2 (263) for Windows (GraphPad Software, Boston, MA, USA). Normality of data distribution was assessed by a Lilliefors-corrected Kolmogorov–Smirnov test. To compare two groups of data, the two-tailed unpaired Student’s *t*-test was used for normally distributed data; otherwise, the Mann–Whitney U test was applied. Multiple groups of data were compared using a one-way ANOVA with Bonferroni correction. For paired data (measurements from the same muscles before and after treatment), a two-tailed paired *t*-test was employed. Statistical significance is denoted in the graphs as follows: NS, not significant; *, *p* < 0.05; **, *p* < 0.01; ***, *p* < 0.001; #, *p* < 0.0001; a threshold of *p* < 0.05 was considered statistically significant. The graphs show the means ± SEM.

Further ARRIVE 2.0 elements, including details of experimental design, sample sizes, and statistical methods, are provided in the corresponding Results sections and Figure legends below.

## 3. Results

### 3.1. LTX^N4C^ Causes Bursts of High-Frequency Neurotransmitter Release at the Mouse NMJ

We previously reported that LTX^N4C^ induces strong “spontaneous” quantal ACh release at the NMJ [15]. Here, we undertook an in-depth study of this effect. Presynaptic exocytotic events—instances of ACh release, typically referred to as exocytosis—were accurately detected as individual MEPPs via electrophysiological recordings of postsynaptic V_m_. This approach enabled us to probe the presynaptic mechanisms activated by LTX^N4C^—mechanisms that reflect vesicle fusion events and ACh release.

**Figure 1:**
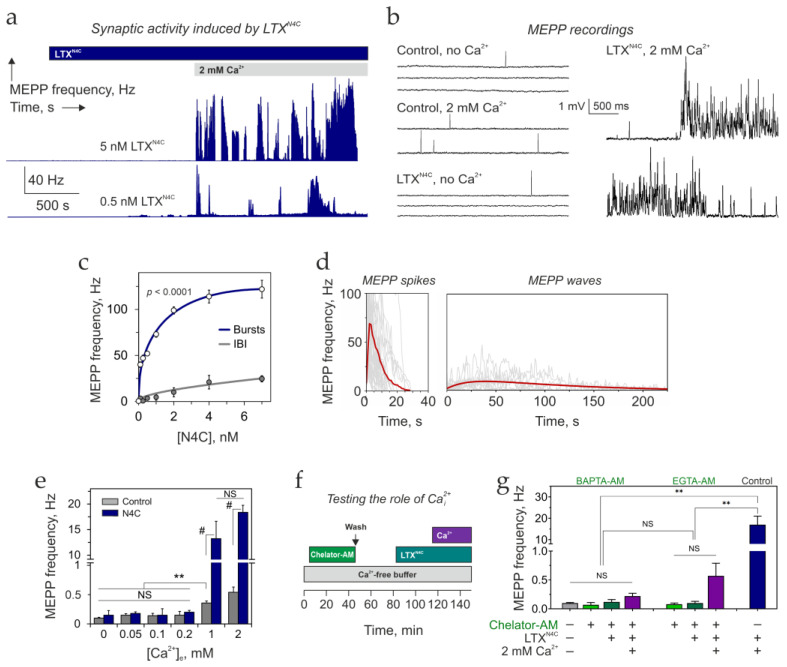
LTX^N4C^ induces high-frequency, Ca^2+^_cyt_-dependent ACh exocytosis at the mouse NMJ. (**a**) Synaptic activity triggered by LTX^N4C^ at different concentrations, in the absence or presence of Ca^2+^_e_. MEPP frequencies are shown in 1 s bins; toxin and Ca^2+^ additions are indicated by bars above the traces. (**b**) Representative postsynaptic V_m_ recordings under the indicated conditions (see Section 2.3 for V_m_ details). Individual MEPPs appear as upward deflections. Note the low basal MEPP frequency under control conditions and the burst of high-frequency MEPPs upon addition of both LTX^N4C^ and Ca^2+^. (**c**) LTX^N4C^ dose–response relationship for MEPP frequency during bursts and IBIs. The two curves differ significantly at [LTX^N4C^] ≥ 0.1 nM (*p* < 0.0001; FANOVA). (**d**) Overlaid MEPP frequency plots of individual bursts induced by 1 nM LTX^N4C^ (gray), grouped as “MEPP spikes” or “MEPP waves.” Red lines indicate averaged plots. (**e**) Mean MEPP frequency in control and LTX^N4C^-treated NMJs as a function of [Ca^2+^_e_]. (**f**) Schematic of the intracellular chelator loading experiments. Concentrations: BAPTA-AM (500 μM) or EGTA-AM (1 mM); LTX^N4C^, 0.25 nM; Ca^2+^_e_, 2 mM. (**g**) Mean MEPP frequencies under each condition of the chelator loading experiments. Symbols indicate statistical significance: **, *p* < 0.01; #, *p* < 0.0001; NS, not significant. *n* = 6, *N* = 25–36 in (**e**); *n* = 3, *N* = 18 in (**g**).

We first examined the toxin’s effect in the presence or absence of Ca^2+^_e_ (Figure 1). In agreement with previous findings [12], although LTX^N4C^ binds its receptor LPHN1 in Ca^2+^-free conditions, it failed to elicit any effect even after prolonged incubation of neuromuscular preparations in 0 Ca^2+^_e_ (Figure 1a). However, upon addition of 2 mM Ca^2+^, the effect of 0.25 nM LTX^N4C^ developed immediately. MEPP frequency increased ~50-fold compared to basal frequency in 2 mM Ca^2+^_e_ alone (17.5 ± 2.07 Hz, *n* = 32, vs. 0.36 ± 0.03 Hz, *n* = 25) (Figure 1b).

Most strikingly, LTX^N4C^-induced exocytosis was not uniform in frequency. Instead, it consisted of irregular, short bursts of very high-frequency synaptic activity interspersed with longer periods of moderate release, IBIs (Figure 1a,b). Both burst and IBI frequencies increased with toxin concentration, reaching 100–150 Hz during bursts and 20–25 Hz during IBIs (Figure 1c). At 0.25 nM LTX^N4C^—the concentration used throughout this study—the average frequency was 47.4 ± 4.09 Hz (*n* = 33) within bursts and 1.2 ± 0.10 Hz (*n* = 33) during IBIs.

Burst duration was also highly variable, ranging from 200 ms to hundreds of seconds. Bursts could be further subdivided into two types: brief, high-frequency *spikes* and longer, slower *waves* of synaptic activity (Figure 1d). On average, MEPP spikes lasted 29.1 ± 9.9 s, while MEPP waves continued for 220 ± 18.3 s. IBIs varied from 0.6 s to 23 min, with an average duration of 143.5 ± 54.8 s.

The distribution of MEPP frequencies during LTX^N4C^-induced activity (Figure S1a) revealed that IBIs themselves contained two distinct patterns: (i) intervals of basal activity (comparable to control conditions without LTX^N4C^, ~0.36 Hz) and (ii) intervals of slow, *tidal* increases in MEPP frequency (1–3 Hz). Because the onset and offset of these tidal intervals were difficult to delineate, we quantified them as part of the IBIs—despite their significant contribution to the toxin’s overall effect, as described later.

Crucially, MEPPs evoked during bursts and IBIs were indistinguishable from spontaneous MEPPs in amplitude and duration (Figure S1b,c). This indicates that LTX^N4C^ acts presynaptically to increase the probability of exocytosis, without detectable postsynaptic effects. Nevertheless, LTX^N4C^-induced exocytosis was clearly asynchronous. Coincident MEPPs—adding up to 5–15 times the unitary amplitude—were observed only at the onset of MEPP spikes (Figure S1d) and very rarely reached the V_m_ threshold for triggering a muscle action potential. We interpret this as stochastic coincidence of high-frequency exocytosis from independent release sites within a given NMJ, rather than synchronous, multiquantal release.

A second defining feature of LTX^N4C^-evoked ACh exocytosis was its strict dependence on Ca^2+^_e_ (Figure 1e). Bursts of MEPPs appeared only when Ca^2+^_e_ was equal to or exceeded 1 mM, while at 0.2 mM Ca^2+^_e_, no characteristic activity was observed. If 1–2 mM Ca^2+^ was added after LTX^N4C^, bursting activity consistently began within 1–2 min, a delay likely owing to Ca^2+^ diffusion. In contrast, when LTX^N4C^ was added to preparations already bathed in 2 mM Ca^2+^, its effects developed only after a considerable delay (Figure S1e). This lag was inversely proportional to toxin concentration (Figure S1f): at 0.25 nM LTX^N4C^, the lag period averaged 17 ± 2.5 min (*n* = 5), whereas 7 nM LTX^N4C^ caused an almost immediate effect. Once initiated, the activity was indistinguishable whether Ca^2+^ was added before or after toxin.

Because the toxin’s effect strictly required Ca^2+^_e_, we asked whether Ca^2+^_e_ acts exclusively extracellularly or whether it must enter nerve terminals. To distinguish these possibilities, we preloaded nerve terminals with the membrane-permeable Ca^2+^ chelator BAPTA-AM (applied at 500 µM), then added 0.25 nM LTX^N4C^ and 2 mM Ca^2+^_e_ (Figure 1f). Cytosolic BAPTA suppressed unstimulated synaptic activity below even control levels (Figure 1g and Figure S1g), consistent with a constitutive role for cytosolic Ca^2+^ in spontaneous exocytosis. More importantly, LTX^N4C^ was unable to evoke its characteristic bursting activity under these conditions, despite the presence of Ca^2+^_e_. Compared to the control conditions (0.25 nM LTX^N4C^, 2 mM Ca^2+^_e_), the inclusion of the intracellular BAPTA produced a 99.95% ± 3.40% inhibition of the LTX^N4C^-induced effects (Table 1).

Although this result was unequivocal, the widespread use of intracellular BAPTA to study Ca^2+^_cyt_ has recently been criticized for potential off-target effects, such as ATP depletion, cytoskeleton disassembly, and inhibition of IP_3_Rs [40,41]. To control for non-specific actions of BAPTA and isolate chelation-dependent mechanisms, we conducted a similar experiment using EGTA-AM, a membrane-permeable chelator with a comparable affinity for Ca^2+^ [42]. Given its different structure, EGTA is unlikely to exhibit the same off-target effects as BAPTA [41]. Although EGTA has much slower Ca^2+^ binding kinetics than BAPTA and often fails to inhibit fast Ca^2+^-dependent processes [42], it has been successfully used as an intracellular chelator when the source of Ca^2+^_cyt_ and its sensor are separated by more than 30 nm [43,44]. In our experiments, intracellular EGTA inhibited the LTX^N4C^-induced bursts of quantal release by ~95% on average (Figure 1g and Figure S1h; Table 1). Notably, this inhibition was not complete, and some NMJs exhibited sudden, very short bursts of high-frequency MEPPs (up to ~50 Hz; Figure S1h).

Together, these observations support several important conclusions:

1. LTX^N4C^ acts presynaptically to induce bursts of ACh exocytosis;
2. This action requires an increase in Ca^2+^_cyt_ in nerve terminals;
3. The irregular, burst-like pattern of exocytosis reflects imperfect periodicity in the nerve terminal’s readiness to respond to the toxin;
4. The delay preceding LTX^N4C^ action in the continuous presence of Ca^2+^_e_ suggests a multistep signaling pathway downstream of toxin binding;
5. When toxin is added before Ca^2+^_e_, it likely primes the release machinery, but Ca^2+^ influx remains the obligate trigger for exocytosis under physiological conditions;
6. The two distinct burst morphologies—MEPP spikes and MEPP waves—may reflect different functional states of the underlying signaling machinery.

These possibilities are addressed later in this paper.

### 3.2. LTX^N4C^ Increases Cytosolic Ca^2+^ Levels in Presynaptic Terminals

**Figure 2:**
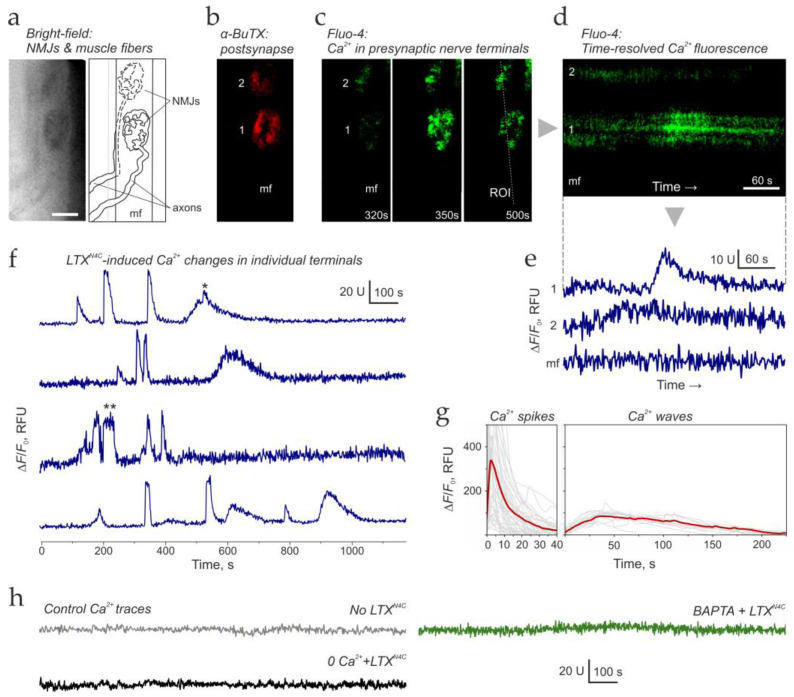
LTX^N4C^ induces spikes and waves of Ca^2+^_cyt_ in presynaptic nerve terminals at the mouse NMJ. Motor nerve terminals were pre-loaded with the Ca^2+^ indicator Fluo-4, stimulated with 0.25 nM LTX^N4C^, and fluorescence was monitored by time-lapse microscopy. (**a**) Left: Bright-field image of two representative NMJs. Scale bar, 20 μm. Right: Corresponding schematic diagram; mf, muscle fiber. (**b**) Fluorescence image of postsynaptic ACh receptors at the same NMJs, labeled with αBuTX Alexa Fluor 546. (**c**) Fluo-4 fluorescence in the same NMJs after application of 0.25 nM LTX^N4C^. The toxin induces a specific increase in Ca^2+^_cyt_ within nerve terminals, with no detectable fluorescence change in muscle fibers. (**d**) Kymograph showing time-resolved Fluo-4 fluorescence along the linear ROI in (**c**). The vertical axis represents pixel positions along the ROI; the horizontal axis represents time. (**e**) Normalized fluorescence (ΔF/F_0_) from the kymograph in (**d**). (**f**) Representative normalized Fluo-4 fluorescence traces from four individual nerve terminals stimulated with LTX^N4C^, showing both sharp Ca^2+^_cyt_ spikes and slower Ca^2+^_cyt_ waves. Note a slow rise in the background Ca^2+^ fluorescence in all traces. Symbols: *, a fluorescence spike superimposed on a wave; **, a series of unresolved fluorescence spikes. (**g**) Overlay of individual LTX^N4C^-induced spikes and waves of Ca^2+^_cyt_ fluorescence (gray), aligned by the onset time. Red lines represent the averaged traces. (**h**) Control Ca^2+^ fluorescence traces demonstrating that Ca^2+^_cyt_ elevations do not occur in nerve terminals that are unstimulated (gray) or stimulated with LTX^N4C^ in the absence of Ca^2+^_e_ (black) or after preloading with BAPTA-AM (green).

To directly test whether LTX^N4C^ alters presynaptic Ca^2+^ dynamics, we monitored Ca^2+^ levels in motor nerve terminals using fluorescence imaging (Figure 2).

First, we conducted experiments aimed at detecting and characterizing any changes in presynaptic Ca^2+^_cyt_ induced by LTX^N4C^. For this purpose, nerve terminals were selectively loaded with the fluorescent Ca^2+^ sensor dye Fluo-4-AM. To avoid dye uptake into muscle cells, the dye was not added directly to the preparation but instead introduced into a suction pipette, which was used to engulf the free end of the medial plantar nerve, carefully detached from the preparation. To facilitate Fluo-4-AM entry into the axoplasm, we added a detergent to the dye solution in the suction pipette to loosen the myelin around the nerve stump. After 3–4 h of incubation, the de-esterified dye diffused through the axoplasm into small nerve branches and some nerve terminals, without entering the associated muscle cells.

Simultaneously with Fluo-4 loading, we incubated muscle preparations with a fluorescent derivative of αBuTX to label postsynaptic ACh receptors. This dual-labeling approach allowed us to identify regions containing multiple NMJs where Fluo-4 staining was restricted to nerve terminals, with no fluorescence in the associated muscle fibers (Figure 2a–c).

We then monitored Fluo-4 fluorescence in these identified regions. In the presence of 2 mM extracellular Ca^2+^ but without LTX^N4C^, no changes in Fluo-4 fluorescence were detected in nerve terminals. However, 10–20 min after adding 0.25 nM LTX^N4C^, most nerve terminals began exhibiting periodic increases in Fluo-4 fluorescence, reflecting elevations and falls in presynaptic Ca^2+^_cyt_ levels (Figure 2d,e). Muscle cells, which contained no Fluo-4 dye, displayed no Ca^2+^_cyt_ changes under these conditions (Figure 2c–e).

Notably, these presynaptic Ca^2+^_cyt_ peaks appeared independently in adjacent synapses and even in individual boutons of a single nerve terminal (Figure 2d,e). This spatial heterogeneity provides direct visual evidence supporting our hypothesis that LTX^N4C^ independently activates distinct release sites within a given terminal.

In some experiments, Fluo-4-AM escaped the suction pipette and flooded the preparation, resulting in both nerve terminals and muscle fibers taking up the dye. In such cases, changes in Ca^2+^_cyt_ fluorescence could not be immediately attributed to either compartment. However, we found that the fluorescent Ca^2+^_cyt_ signal was consistently higher in nerve terminals than in the muscle cells they synapsed on (Figure S2). By appropriately adjusting the fluorescence intensity window, we could resolve terminal-specific Ca^2+^ spikes (Figure S2b) and waves (Figure S2d). Crucially, differential Ca^2+^ fluorescence traces (Figure S2e) revealed that presynaptic Ca^2+^ signals exhibited higher amplitudes and earlier onsets than the postsynaptic Ca^2+^ signals, which were activated secondarily by the firing of the toxin-stimulated motor terminals. Thus, as also described previously [45], even NMJs with both neuronal and muscle cells loaded with Fluo-4 could still be used for selective detection of presynaptic Ca^2+^ signals.

Ca^2+^_cyt_ fluorescence recordings from multiple individual nerve terminals revealed irregular patterns of activity, comprising two distinct components: brief, high-amplitude Ca^2+^_cyt_ spikes and slower, low-amplitude Ca^2+^_cyt_ waves (Figure 2f). These Ca^2+^ dynamics closely resembled the bursts of MEPP frequency recorded electrophysiologically (compare Figure 1a and Figure 2f). Furthermore, the average waveforms of Ca^2+^_cyt_ spikes and waves (Figure 2g) were nearly identical to those of MEPP frequency spikes and waves (Figure 1d). Specifically, the spike/wave amplitude ratio was 5.8 ± 1.2 for MEPP bursts and 4.9 ± 2.1 for Ca^2+^_cyt_ changes (not significantly different). Similarly, both datasets showed identical duration (31.5 ± 2.03 s for spikes and 220 ± 10.7 s for waves) and the rate of rise (35 ± 2.2 units/s for spikes and 0.4 ± 0.12 units/s for waves) of respective events.

Such Ca^2+^_cyt_ spikes and waves never appeared in the absence of LTX^N4C^ (Figure 2h), and the fluorescence of all recorded nerve terminals and muscle cells remained stable over the course of the experiment (Figure S1f). Moreover, when terminals were bathed in a Ca^2+^-free buffer or preloaded with BAPTA-AM, LTX^N4C^ failed to induce the characteristic changes in Ca^2+^_cyt_ levels (Figure 2h and Figure S1g).

Given the established dependence of MEPP bursts on Ca^2+^ influx and the striking similarity in their dynamics, these data strongly suggest that LTX^N4C^-induced MEPP bursts are driven by corresponding fluctuations in presynaptic Ca^2+^_cyt_. Furthermore, the toxin causes at least two types of Ca^2+^_e_ influx: brief and intense, producing short, giving rise to Ca^2+^_cyt_ spikes, and long and weak, producing Ca^2+^_cyt_ waves and IBIs. These Ca^2+^ inflows could involve two distinct types of Ca^2+^ channels.

### 3.3. LTX^N4C^ Effects Are Mediated by LPHN1

To understand the mechanism by which LTX^N4C^ stimulates Ca^2+^ influx and neurotransmitter release, we first sought to identify the presynaptic receptor protein(s) mediating its effects.

Similar to its parent molecule αLTX, LTX^N4C^ has been shown to bind two principal neuronal receptors: LPHN1 and NRXN1α [12]. NRXN1α could be excluded as a mediator of the specific LTX^N4C^ actions, because it does not bind LTX^N4C^ in the presence of Ba^2+^ or Sr^2+^ [12], while these cations fully support LTX^N4C^-induced burst-like release [12]. In addition, protein tyrosine phosphatase σ (PTPσ), another neuronal receptor for αLTX [46], could potentially mediate the effect of LTX^N4C^, although their interaction has not been directly tested.

Given the possibility of LTX^N4C^ acting through a variety of neuronal proteins, an important observation provided direct evidence for the role of LPHN1, when LPHN1 gene KO was shown to block LTX^N4C^-induced burst-like exocytosis [47]. Interestingly, LPHN1 KO did not remove all toxin binding sites in central neurons, although the residual toxin binding did not seem to contribute to LTX^N4C^-evoked increase in neurotransmitter release [47]. Therefore, LPHN1 is likely the main mediator of the specific effects of the mutant toxin.

**Figure 3:**
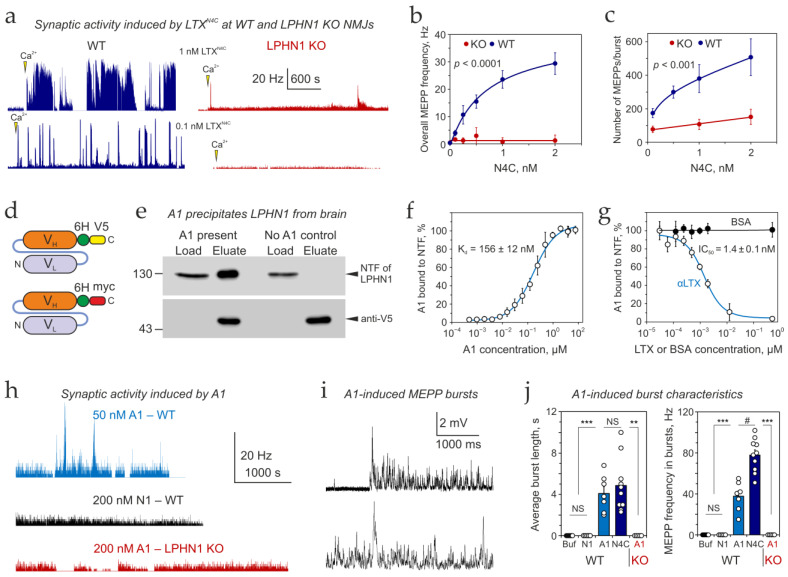
LPHN1 is the primary mediator of LTX^N4C^-induced burst-like exocytosis. (**a**–**c**) LTX^N4C^ effects at the NMJs from WT and LPHN1 KO mice. (**a**) MEPP frequencies induced by LTX^N4C^ in respective NMJs and at specified concentrations. The toxin was added 30 min before the start of recording and 2 mM Ca^2+^ was added as indicated by arrowheads. LTX^N4C^ elicited large numbers of high-frequency bursts in WT NMJs, but only very rare and short bursts of MEPPs in LPHN1 KO preparations. (**b**) Dose–response curves for overall MEPP frequencies elicited by LTX^N4C^, summarizing the data in (**a**) and showing the dramatic increase in MEPP frequency in WT synapses and the lack of an overall increase in LPHN1 KO synapses. The curves are significantly different at LTX^N4C^ concentrations ≥ 0.1 nM (*p* < 0.0001, FANOVA; *n* = 18 for both conditions). (**c**) The mean number of MEPPs within a burst at WT and KO NMJs after stimulation with different LTX^N4C^ concentrations. The curves are significantly different at LTX^N4C^ concentrations ≥ 0.1 nM (*p* < 0.001, FANOVA; *n* = 18 for WT and 3 for KO). (**d**–**g**) The anti-LPHN1 scFv A1 binds to the NTF of LPHN1 and competes with αLTX for the binding site. (**d**) Schematic structure of the scFv antibodies A1-V5 (top) and A1-myc (bottom). V_H_ and V_L_, variable regions of the heavy and light immunoglobulin chains, respectively; V5, myc, and 6H (6 His) are epitopes for immunodetection and purification. (**e**) A1-V5 precipitates LPHN1 from mouse brain lysate. Western blot analysis of input lysate and eluate fractions from affinity chromatography on an anti-V5 mAb column with or without the addition of A1-V5. Blots were probed with an anti-LPHN1 antibody, RL1 (top), and anti-mouse IgG antibody (bottom). (**f**) Binding affinity of A1-myc for the LPHN1 NTF. Increasing concentrations of A1-myc were applied to immobilized recombinant V5-NTF (see Figure S3b for assay schematic, except αLTX was omitted in the binding assay). The calculated *K*_d_ was 156 ± 12 nM. (**g**) αLTX competes with A1 for binding to the LPHN1 NTF. The assay was performed as in (**f**) (see Figure S3b for a diagram), with a fixed concentration of A1-myc incubated in the presence of increasing concentrations of αLTX or BSA as a negative control. (**h**–**j**) The scFv A1 induces ACh exocytosis by acting via LPHN1. (**h**) The A1 antibody mimics the effect of LTX^N4C^. Application of 50 mM A1 induced bursts of MEPPs in WT NMJs, while 200 nM A1 failed to induce bursts in LPHN1 KO NMJs. Application of a control scFv antibody (N1, 200 nM), which does not bind LPHN1, had no effect on WT or KO synapses. (**i**) Representative traces of MEPP bursts recorded from a WT NMJ following application of A1. (**j**) Quantitative analysis of A1-induced bursts. The graphs show the mean burst duration (left) and mean MEPP frequency within bursts (right) in A1-treated WT NMJs. A1 did not induce bursts in LPHN1 KO NMJs, and N1 did not induce bursts in any NMJs. Statistical significance is indicated by symbols: **, *p* < 0.01; ***, *p* < 0.001; #, *p* < 0.0001; NS, non-significant; *n* = 6 per condition.

To ascertain the critical role of this receptor in the LTX^N4C^ actions, first, we determined the concentration-dependence of LTX^N4C^-induced increase in MEPP frequency at the NMJs from WT and LPHN1 KO mice (Figure 3a, blue and red, respectively). In line with the previous results on LPHN1 KO preparations [47], the basal frequency of exocytosis in LPHN1 KO was significantly higher than in WT preparations (1.7 ± 0.3 Hz, *n* = 6 vs. 0.37 ± 0.05, *n* = 13). Addition of LTX^N4C^ to WT preparations caused a massive and concentration-dependent increase in the frequency of exocytosis, reaching 4.9 ± 0.8 Hz at 0.25 nM (*n* = 6) and exceeding 30 Hz (30 ± 4.8 Hz, *n* = 4) at 2 nM LTX^N4C^ (Figure 3b, blue). By contrast, when the same concentrations of LTX^N4C^ were added to LPHN1 KO preparations, they did not significantly change the overall frequency of exocytotic events (1.8 ± 0.5 Hz, *n* = 6 at 0.25 nM LTX^N4C^) (Figure 3b, red).

Surprisingly, LTX^N4C^-treated KO preparations did show occasional low-frequency bursts of MEPPs. However, these episodes were rare and brief, and contained 3–5-fold fewer MEPPs than in respective WT preparations (Figure 3c). Also, while the number of MEPPs per burst strongly depended on LTX^N4C^ concentration at WT synapses, a 20-fold increase in the toxin concentration at KO NMJs did not significantly change MEPP frequency. This residual activity, while minimal, suggests a secondary, low-efficiency pathway for LTX^N4C^ action that is independent of LPHN1 (see Section 4.1).

Second, we took advantage of another useful tool created in our laboratory: a recombinant anti-LPHN1 scFv antibody A1 (Figure 3d–j), which was isolated from a phage-display library by multiple rounds of selection against the NTF of LPHN1. Two versions of the A1 antibody were produced, supplemented with either a V5 or a myc immunological tag (Figure 3d). A1 specifically binds LPHN1 and precipitates it from solubilized mouse brain (Figure 3e) [48]; in an experiment schematically presented in Figure S3a. Furthermore, sucrose density gradient centrifugation experiments (Figure S3d,e) demonstrate that A1 binds and sediments in a complex with the NTF. A LPHN1 binding assay (diagrammatically explained in Figure S3b) was used to determine the A1 affinity for LPHN1, which equaled 156 ± 12 nM (Figure 3f).

Interestingly, we found that αLTX competes with A1 for LPHN1 binding (Figure 3g), although the toxin shows a 100-fold higher affinity for LPHN1 (*IC*_50_ = 1.4 nM). Control experiments confirmed that this competition was not due to a direct interaction between αLTX and the A1 antibody itself (Figure S3c). This indicates that αLTX and A1 bind to the same locus on the NTF of LPHN1 and compete for it.

Given that A1 mimics the αLTX-LPHN1 interaction, we hypothesized that A1 might similarly activate the receptor to induce exocytosis. To test this, we applied A1 to mouse NMJs. Strikingly, A1 did induce high-frequency bursts of MEPPs at WT mouse NMJs, but not at NMJs from LPHN1 KO mice (Figure 3h). A control scFv antibody N1, which was isolated from the same phage display library but did not bind LPHN1, was unable to induce any increase in synaptic activity (Figure 3h). This confirms that the effect of A1 is specifically due to its interaction with LPHN1, rather than a non-specific effect of antibody application or the scFv scaffold. The A1-induced bursts were similar to those induced by LTX^N4C^ both in terms of morphology (Figure 3i) and duration (Figure 3j, left), although attaining a lower average MEPP frequency (Figure 3j, right). This finding strongly supports the hypothesis that LPHN1 is a central molecule for ligand-induced burst-like increase in synaptic activity at the mouse NMJ.

These results led us to the following conclusions:

1. LPHN1 is the main mediator of LTX^N4C^ effects on ACh exocytosis;
2. Ligand binding to a specific site on the NTF of LPHN1 is responsible for the characteristic increase in synaptic activity;
3. In addition to its main action via LPHN1, LTX^N4C^ could activate a low-efficiency alternative—or complementary—signaling pathway.

While these conclusions indicated a pivotal role for LPHN1 in LTX^N4C^-induced bursts of synaptic activity, they also suggested that this receptor must be localized to motor nerve terminals. Therefore, we decided to investigate the expression and localization of LPHN1 at the NMJ.

### 3.4. LPHN1 Is Presynaptic at the Mouse and Frog NMJ

To map LPHN1 distribution within the NMJ, we first assessed its mRNA expression among the three cellular components of the NMJ: motor neurons, Schwann cells, and muscle fibers. Because presynaptic proteins are translated in motor neuron cell bodies located in the SCVH and then transported to nerve terminals, we measured Lphn1 mRNA levels in microdissected SCVH tissue. To evaluate Schwann cell contribution, we examined the tibial nerve—a branch of which innervates the FDB muscle used in our electrophysiological experiments—as myelinating Schwann cells constitute the predominant cell type in this nerve preparation. Finally, because muscle fibers represent the majority of cell volume and protein synthesis activity in muscle tissue, any Lphn1 mRNA detectable in whole muscle preparations would likely originate from muscle fibers.

**Figure 4:**
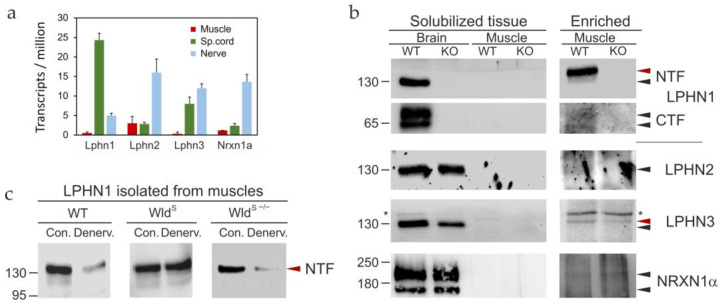
LPNH1 is predominantly expressed in motor nerve terminals. (**a**) RT-qPCR analysis of Lphn1–3 and Nrxn1a mRNA levels in the three cellular compartments contributing to the NMJ structure: spinal cord ventral horn (motor neuron cell bodies), tibial nerve (Schwann cells), and whole muscle (muscle fibers). The data are the means ± SEM; *n* = 3; see text for numerical values. (**b**) Western blot analysis of LPHN1–3 and NRXN1α protein levels in mouse brain and muscle. Left, solubilized membranes from the indicated tissues were probed directly with specific antibodies. LPHN1 is absent from LPHN1 KO brain, confirming antibody specificity. In whole muscle lysates, LPHN1, LPHN2, and NRXN1α are undetectable, while LPHN3 appears as a faint band in WT samples. Right, toxin binding proteins were enriched from muscle lysates by αLTX affinity chromatography prior to Western blotting. After enrichment, LPHN1 is clearly detectable in WT muscle, and LPHN3 becomes more prominent. Red arrowheads indicate proteins with NMJ-specific post-translational modifications, resulting in a slightly decreased mobility; asterisk, non-specific protein. Molecular mass markers (kDa) are indicated on the left. (**c**) Denervation experiments confirm the neuronal origin of LPHN1 detected in muscle preparations. LPHN1 was isolated from control innervated (Con) and denervated (Denerv.) muscles by αLTX affinity chromatography and detected by Western blotting. In WT mice (left), denervation sharply reduces LPHN1 levels. In Wld^S^ mice (center), which undergo slow Wallerian degeneration, axotomy causes no detectable LPHN1 loss. In Wld^S^ protein KO (Wld^S−/−^) mice (right), denervation again reduces LPHN1 levels. These data demonstrate that LPHN1 is synthesized in motor neurons and transported to nerve terminals via motor axons.

Using RT-qPCR, we compared mRNA levels of LPHN1–3 and NRXN1α—four receptors relevant to αLTX studies—across these tissues (Figure 4a). The results revealed striking differences in expression patterns. Remarkably, 81.6 ± 5.91% of all Lphn1 mRNA was detected in the SCVH, with 16.8 ± 1.94% present in the tibial nerve and only 1.68 ± 0.97% (essentially at the detection limit) in muscle. This neuronal enrichment was unique to Lphn1; the other receptor mRNAs were predominantly (59–85%) expressed in the nerve, likely within Schwann cells. SCVH also showed appreciable Lphn3 expression (39.3 ± 8.52% of its mRNA), while Lphn2 and Nrxn1a were only moderately expressed in this compartment (13.0 ± 2.02% and 14.7 ± 3.67%, respectively). Lphn2 mRNA was also detectable in muscle fibers (13.7 ± 7.93%). These data establish LPHN1 as the most neuronally enriched receptor in this group, positioning it as the primary candidate for mediating the effects of LTX^N4C^—particularly given its highest affinity for αLTX [34,48].

We next asked whether this mRNA distribution translated into corresponding protein expression patterns. Immunoblotting of solubilized mouse brain from WT and LPHN1 KO mice confirmed antibody specificity: all four proteins were readily detected in WT brain, and—with the expected exception of LPHN1—in KO brain lysates (Figure 4b). However, when we probed whole muscle preparations, none of the four receptors were directly detectable, with only a marginal LPHN3 signal appearing in WT samples (Figure 4b). This suggested two conclusions: these receptors are not abundantly expressed in muscle fibers, and their expression is confined to a small compartment within the nerve-muscle preparation—most likely the NMJ itself.

To test the latter hypothesis, we enriched synaptic proteins from mouse brain extracts using αLTX affinity chromatography. This procedure allows one to isolate from tissue lysates all receptors, including LPHN1–3 and NRXN1α, that bind αLTX and LTX^N4C^. Following enrichment, LPHN1 was abundantly detected in WT synaptic preparations, and LPHN3 became clearly visible in both WT and KO enriched samples (Figure 4b). In contrast, LPHN2 and NRXN1α remained barely detectable even after enrichment. These findings align with the RT-qPCR data if we interpret them as follows: LPHN1 and LPHN3 are expressed in motor neurons, while LPHN2 and NRXN1α derive predominantly from Schwann cells—both populations representing such a small fraction of a whole nerve-muscle preparation that their protein products escape detection without prior enrichment.

Interestingly, the NMJ-associated forms of LPHN1 and LPHN3 migrated at a slightly higher relative molecular mass than their brain-derived counterparts (red arrowheads in Figure 4b), suggesting that these proteins undergo NMJ-specific post-translational modifications.

**Figure 5:**
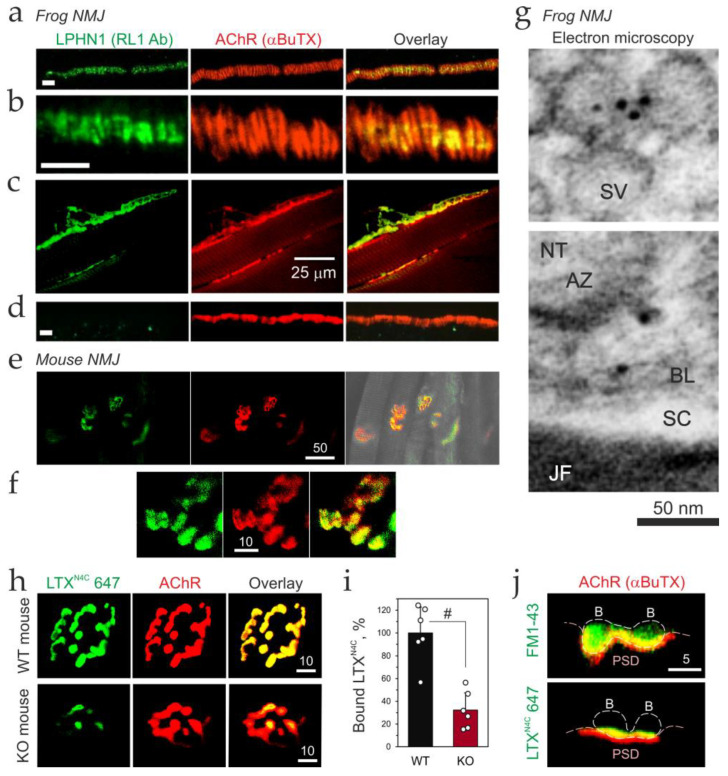
LPHN1 localizes to presynaptic nerve terminals at the frog and mouse NMJ. (**a**–**d**) Localization of LPHN1 at the frog NMJ by fluorescent confocal microscopy. Preparations were labeled for postsynaptic AChRs with rhodamine-conjugated αBuTX (red), permeabilized and immunostained for LPHN1 (green, affinity-purified RL1 antibody), followed by FITC-conjugated anti-rabbit IgG. (**a**) Low-magnification top view of an NMJ. Scale bar, 10 µm. (**b**) High-magnification top view. Scale bar, 10 µm. LPHN1 immunoreactivity appears in bands juxtaposed to the postsynaptic AChR-rich ridges. (**c**) Lateral (xz) view of an NMJ. Scale bar, 25 µm. LPHN1 labeling is strictly presynaptic, with some signal associated with synaptic vesicle clusters within boutons and axon branches. (**d**) Following physical removal of the motor nerve terminal after treatment with collagenase, LPHN1 immunoreactivity is abolished while AChR staining persists. Scale bar, 10 µm. (**e**,**f**) Localization of LPHN1 at the mouse NMJ. The same methodology and color scheme as in (**a**–**d**) was applied, except αBuTX was conjugated to Alexa Fluor 546 (red), and LPHN1 was detected using an Alexa Fluor 488-conjugated secondary antibody (green). (**e**) Low-magnification image of a bundle of muscle fibers from the FDB muscle, showing multiple NMJs and associated axons. Scale bar, 50 µm. (**f**) High-magnification top view of a mouse NMJ. LPHN1 is enriched in presynaptic boutons directly apposed to the αBuTX-labeled postsynaptic specializations. Scale bar, 10 µm. (**g**) Immunogold labeling on LPHN1 in the frog NMJ using the affinity-purified RL1 antibody (see Figure S4b for lower-magnification images). Top, LPHN1 in presynaptic vesicles; bottom, LPHN1 labeled on the presynaptic membrane near an active zone (AZ). Arrows point at gold particles; AZ, active zone; BL, basal lamina; JF, junctional folds (muscle); SC, synaptic cleft; SV, synaptic vesicles. Most LPHN1 labeling is found in the motor terminal, with only background staining of the muscle. (**h**–**j**) Binding of fluorescent LTX^N4C^ is LPHN1-dependent at the mouse NMJ. Non-permeabilized mouse FDB preparations were co-incubated with αBuTX-Alexa Fluor 546 (red) and LTX^N4C^ conjugated to Alexa Fluor 647 (green), and imaged in situ by confocal microscopy. (**h**) Top-view images show abundant LTX^N4C^ binding at NMJs from WT mice (top panels), which is markedly reduced, but not fully eliminated, at NMJs from LPHN1 KO mice (bottom panels). Scale bars, 10 µm. (**i**) Quantification of the data in (**h**). Fluorescence intensity was measured under identical conditions and normalized to the αBuTX signal. Statistical significance: #, *p* < 0.0001; *n* = 6 NMJs. (**j**) Cross-sectional views of WT NMJs stained with the endocytotic dye FM1-43 (left) or with fluorescent LTX^N4C^ (green) and αBuTX (red) as in (**h**). Z-stack images were used for 3D reconstruction and the generation of cross-sections. The dotted lines delineate the motor nerve terminal (white) and the muscle fiber membrane (pink). B, boutons; PSD, postsynaptic density. Scale bar, 5 µm; *n* = 4 per condition.

To directly test whether LPHN1 localizes to motor nerve terminals, we performed denervation experiments. Transection of the sciatic nerve in WT animals triggers Wallerian degeneration [49], wherein the distal portion of motor axons—including nerve terminals—disintegrates and is cleared by macrophages. Forty-eight hours after axotomy, the amount of LPHN1 isolated from WT nerve-muscle preparations using immobilized αLTX dropped sharply by approximately 70% (Figure 4c, WT). To confirm that this loss resulted specifically from distal axonal degeneration, we repeated the experiment in Wallerian degeneration slow (Wld^S^) mice [50], which carry an autosomal-dominant mutation creating a gain-of-function fusion protein that dramatically delays Wallerian degeneration [51]. As predicted, nerve injury in Wld^S^ mice caused no detectable decrease in LPHN1 levels (Figure 4c, Wld^S^). As control, we examined Wld^S−/−^ mice, in which the chimeric Wld^S^ gene was bred out, restoring normal Wallerian degeneration. In these animals, nerve injury again produced a marked reduction in LPHN1 levels (Figure 4c, Wld^S−/−^). The residual LPHN1 detectable in denervated muscle preparations likely reflects partial release of its NTF into the synaptic cleft (see Figure 5 below), where it remains for several days. Together, these results strongly suggest that LPHN1 is synthesized in motor neurons and transported to nerve terminals via motor axons.

Having established LPHN1 as a motor neuron-derived protein, we sought to determine its precise sub-synaptic localization. We turned to the frog NMJ, an ideal preparation for such ultrastructural studies due to its large size, regular organization of active zones, and experimental accessibility. An affinity-purified anti-LPHN1 antibody recognized two bands in solubilized frog brain membranes, corresponding to full-length LPHN1 (~190 kDa) and its NTF (~130 kDa) (Figure S4). Preincubation of the antibody with the GST-NTF fusion protein (used in affinity purification) eliminated the specific signals.

We examined LPHN1 localization by confocal fluorescence microscopy of teased cutaneous pectoris nerve-muscle preparations. NMJs were identified by staining ACh receptors (AChRs) with fluorescent αBuTX (Figure 5a,b; red). Top views revealed AChR clusters forming regularly spaced bands, located perpendicular to the NMJ long axis at ~1-μm intervals and corresponding to the postsynaptic folds. Strikingly, anti-LPHN1 immunoreactivity (green) displayed a nearly identical banding pattern, with LPHN1 bands also spaced at ~1-μm intervals and closely aligned with AChR clusters (*n* = 12 NMJs; Figure 5a,b). However, consistent with a presynaptic localization, the LPHN1 bands were always shorter than the underlying postsynaptic folds—a pattern characteristic of active zones positioned on the presynaptic membrane directly opposite postsynaptic AChR clusters. This organization was observed in all preparations examined (5 muscles, 52 nerve terminals).

Lateral views of doubly stained NMJs provided even clearer evidence of presynaptic LPHN1 localization (Figure 5c). LPHN1 was especially concentrated in the presynaptic membrane facing the postsynaptic specializations, with additional staining visible in synaptic vesicle clusters inside terminals and in motor axons (Figure 5c). These images revealed a distinct separation between LPHN1 and AChR localization. To confirm this interpretation, we physically removed nerve terminals after collagenase treatment. Following this manipulation, all LPHN1 immunoreactivity disappeared, while postsynaptic AChR staining remained intact (Figure 5d).

We then applied the same methodology to mouse nerve-muscle preparations (Figure 5e,f). Although mouse NMJs are smaller than frog NMJs and do not display the same active zone patterning, LPHN1 immunostaining was also found in motor nerve terminal boutons, where it closely apposed—but did not precisely co-localize with—postsynaptic AChR staining (Figure 5f). Low-level LPHN1 immunostaining was also observed in axons (Figure 5e), consistent with protein delivery from motor neuron cell bodies to nerve terminals.

Finally, for precise subcellular localization of LPHN1, we used immunoelectron microscopy of frog NMJs labeled with the affinity-purified anti-LPHN1 antibody and immunogold particles (Figure 5g and Figure S4b). Gold label was observed predominantly in motor nerve terminals, where it concentrated on synaptic vesicles and on the presynaptic plasma membrane. Some label was also present in the synaptic cleft, associated with the basal lamina, suggesting that a portion of the LPHN1 NTF may be released from the nerve terminal membrane. Only background labeling was observed over muscle cells. Together, these results unequivocally establish that LPHN1 localizes to the presynaptic nerve terminal at the frog NMJ.

We next asked whether LTX^N4C^ binds to the same sites on motor nerve terminals identified by anti-LPHN1 antibodies. To address this, we visualized LTX^N4C^ binding sites in WT NMJs using fluorescently labeled LTX^N4C^ (green), with AChRs labeled by fluorescent αBuTX (red) (Figure 5h). The fluorescent LTX^N4C^ staining pattern (Figure 5h) closely reproduced that of the anti-LPHN1 antibody (Figure 5f).

Interestingly, although fluorescent LTX^N4C^ binding to LPHN1 KO NMJs was greatly reduced compared to WT terminals, it was not abolished (Figure 5h,i). Furthermore, the residual LTX^N4C^ binding to KO terminals, albeit weaker and narrower, showed spatial localization similar to that in WT terminals (Figure 5h). This indicates that another LTX^N4C^-binding protein, restricted to the presynaptic membrane of motor nerve terminals, persists in the absence of LPHN1. This protein might mediate the rare bursts of synaptic activity induced by LTX^N4C^ in preparations from LPHN1 KO mice (Figure 3a,c).

To better resolve the closely apposed pre- and postsynaptic compartments, which are indistinguishable in top views, we generated 3D reconstructions from Z-stacks and produced cross-sectional views of LTX^N4C^-labeled NMJs (Figure 5j, bottom). For comparison of the LTX^N4C^ signal with bouton morphology and synaptic vesicle localization, we created similar reconstructions of WT muscle stained by depolarization in the presence of FM1-43, a dye that labels synaptic vesicles by endocytosis [38] (Figure 5j, top). It can be seen that αBuTX staining of the postsynaptic membrane (red) delineates the overall invagination of the postsynaptic membrane beneath motor nerve terminal boutons (postsynaptic junctional folds are not resolved at this resolution). Remarkably, the cross-sections clearly demonstrate that LTX^N4C^ binding (green) concentrates along the presynaptic membrane facing the synaptic cleft, with only slight extension into the bouton cytoplasm (Figure 5j, bottom). By contrast, FM-dye-stained synaptic vesicles (green) occupy a wider space inside the bouton, though they also concentrate near active zones (Figure 5j, top).

Together, these findings demonstrate that LPHN1 localizes to the presynaptic membrane at NMJs from different species, suggesting a conserved distribution and function. Further ultrastructural details will require electron microscopy, but light microscopy already indicates that LPHN1 is most concentrated on the presynaptic membrane but also appears in synaptic vesicles (presumably for axonal transport and storage within boutons).

### 3.5. LPHN1 Acts Mainly via the G_αq_/11 Signaling Pathway

The results described above show that LTX^N4C^ causes a dramatic burst-like increase in the rate of exocytosis by acting via the presynaptic receptor LPHN1. How does this receptor transmit the signal from LTX^N4C^ to ACh exocytosis? LPHN1 is a GPCR, and it is likely to mediate intracellular signaling by activating G proteins. We showed previously that in a model system comprising mouse neuroblastoma cells expressing full-size LPHN1 or its signaling-disabled mutant, LTX^N4C^ causes Ca^2+^ influx by stimulating G_αq_, PLC, and release of Ca^2+^ from intracellular stores [32]. However, only fragmentary information exists about the molecular mechanisms underpinning these effects in neuronal nerve terminals [6], and no explanation has been proposed so far for the mechanism of burst generation.

**Figure 6:**
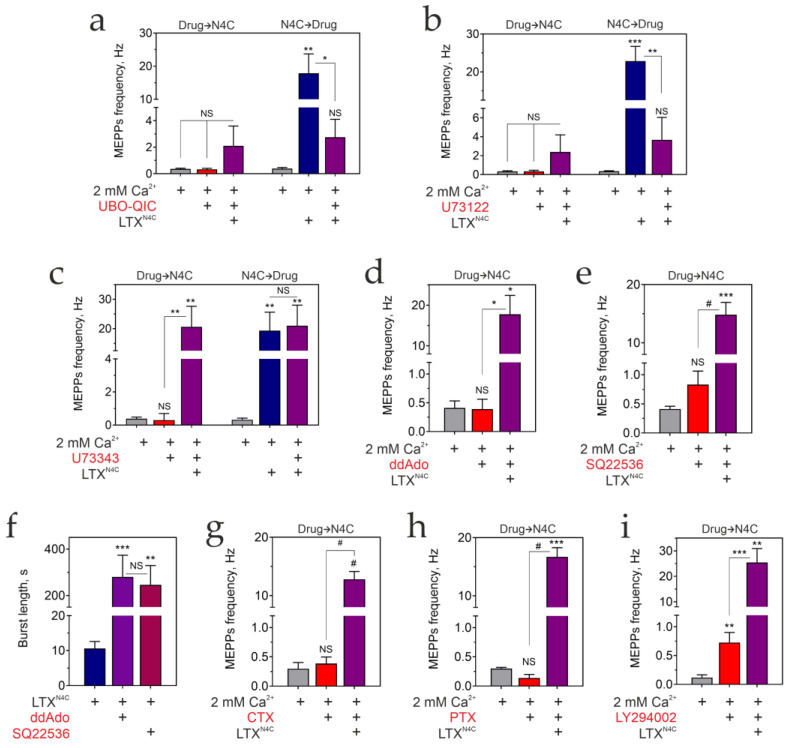
G_αq_ signaling is required for LTX^N4C^-induced, LPHN1-mediated bursts of exocytosis. WT mouse nerve-muscle preparations were incubated in 2 mM Ca^2+^_e_ and stimulated with 0.25 nM LTX^N4C^ before or after treatment with the indicated inhibitors (see Figure S5 for protocol details). (**a**) The G_αq_ inhibitor UBO-QIC (1 µM) was applied before or after LTX^N4C^ (*n* = 4 independent experiments; *N* = 34–36 NMJs analyzed per condition). (**b**,**c**) The PLC inhibitor U73122 (10 µM) (**b**) or its inactive analog U73343 (10 µM) (**c**) were added before or after LTX^N4C^ (*n* = 5; *N* = 32–38). (**d**,**e**) The adenylyl cyclase inhibitors ddAdo (100 µM) (**d**) and SQ22536 (20 µM) (**e**) did not inhibit LTX^N4C^-induced exocytotic bursts (*n* = 5 and 4; *N* = 52 and 41, respectively). (**f**) Both ddAdo and SQ22536 significantly increased the duration of LTX^N4C^-induced bursts (*n* = 5 and 4; *N* = 52 and 41). (**g**) The G_αS_ activator CTX (5 nM, added 4 h before LTX^N4C^) did not affect the basal MEPP frequency or LTX^N4C^-induced bursts of ACh release (*n* = 3, *N* = 12). (**h**) The G_αi/o_ inhibitor PTX (2–20 nM, added 5 h before LTX^N4C^) did not inhibit LTX^N4C^ actions (*n* = 5; *N* = 43). (**i**) The PI_3_Kγ inhibitor LY294002 (30 μM) did not inhibit LTX^N4C^ actions (*n* = 5; *N* = 25). Symbols next to bars indicate statistical significance compared to respective controls, other comparisons are shown by lines: *, *p* < 0.05; **, *p* < 0.01; ***, *p* < 0.001; #, *p* < 0.0001; NS, not significant.

First, in a series of experiments, we confirmed the critical role of G proteins in the LTX^N4C^ action via LPHN1. LPHN1 has been shown to bind G_αq/11_ and G_αo_, but not G_αi_ or G_αz_, while G_αs_ was not tested [5]. Therefore, we compared LTX^N4C^-induced stimulation of the mouse NMJ in a Ca^2+^-containing buffer in the absence (Figure 6a) and presence of specific inhibitors of different Gα proteins or their downstream effectors (Figure 6b–h).

The involvement of G_αq/11_ was probed using UBO-QIC, a cyclic depsipeptide and a specific inhibitor of G_αq/11_ that acts by blocking the GDP/GTP exchange required for G protein activity [52,53]. UBO-QIC prevented LPHN1-mediated bursting activity when added before LTX^N4C^ (Figure 6a and Figure S5b; Table 1) and quickly inhibited it when added after the LTX^N4C^ effects had started (Figure 6a and Figure S5c; Table 1). No differences were found in the average MEPP amplitudes between the conditions (Figure S5a–c), suggesting the effects on frequency are not due to postsynaptic modulation. Interestingly, while UBO-QIC blocked all bursting activity normally elicited by LTX^N4C^, the peptide did not fully inhibit the increased MEPP frequency, corresponding to IBIs in control stimulations (Figure 6a). These results indicate that G_αq_ is required for the generation of bursts, although a distinct, G_αq_-independent mechanism may contribute to the elevated rate of secretion observed during IBIs.

To confirm that this signaling pathway relies on the canonical G_αq_ effector, we used an inhibitor of PLC, U73122 [54,55,56], which prevents the hydrolysis of phosphatidylinositol 4,5-bisphosphate to inositol 1,4,5-trisphosphate (IP_3_) and diacylglycerol. When added before LTX^N4C^, U73122 completely prevented the appearance of the LPHN1-mediated bursts (Figure 6b and Figure S5d; Table 1). Reciprocally, when introduced after the LTX^N4C^ effect had developed, U73122 caused a rapid cessation of bursting activity (Figure 6b and Figure S5e). The average MEPP amplitudes did not significantly change between the experimental conditions. To control for any off-target effects of U73122, we also applied its inactive analogue, U73343 [57,58,59]. Added before or after LTX^N4C^, U73343 had no effect on the LTX^N4C^-induced, LPHN1-mediated bursts of exocytosis (Figure 6c; Table 1). These results, together with the UBO-QIC data, firmly establish that LPHN1 acts via G_αq_ and its downstream effector PLC to generate bursts of quantal ACh release.

Next, we assessed the possible role of G_αs_, which normally activates adenylyl cyclase, increasing cAMP production. Two membrane-permeable adenylyl cyclase inhibitors were tested, 2′,3′-dideoxyadenosine (ddAdo) and 10 μM SQ22536 [60,61]. Neither inhibitor significantly changed the basal frequency of ACh exocytosis in the presence of Ca^2+^_e_ (Figure 6d,e), nor did they prevent the burst-like activity evoked by LTX^N4C^ (Figure 6d,e and Figure S5f,g; Table 1). Strikingly, however, these inhibitors, especially ddAdo, significantly (~20-fold) increased the duration of the toxin-induced bursts (Figure 6f). This result indicates that G_αs_/cAMP signaling is not required for the initiation of bursts. Instead, this pathway appears to play a modulatory role, actively promoting burst termination. Interestingly, both ddAdo and SQ22536 strongly activated spontaneous contractions of muscle fibers, possibly due to prolonged opening of ion channels; however, this postsynaptic effect was not investigated here.

Thus, decreasing cAMP production did not affect the LTX^N4C^-induced actions. To test whether, instead, increasing cAMP levels could play a role, we used CTX, which ADP-ribosylates and activates G_αs_ [62], eventually leading to its downregulation [63]. We found that CTX also did not affect either the resting MEPPs frequency or the LTX^N4C^-induced bursts of exocytosis (Figure 6g and Figure S5h; Table 1).

The involvement of the G_αi/o_ family proteins in LTX^N4C^ actions was then examined. G_αi_ primarily inhibits the cAMP-dependent pathway by reducing adenylyl cyclase activity. G_αo_ is involved in many cellular processes, primarily by inhibiting/activating ion channels, and may also inhibit adenylyl cyclase, although this signaling pathway is not its primary target [64]. The G proteins of the i/o family are sensitive to ADP-ribosylation by PTX [65,66], which was then used in our experiments at concentrations that only affect Gα proteins [66]. However, PTX did not affect either the basal or LTX^N4C^-induced MEPP frequency (Figure 6h and Figure S5i; Table 1), indicating that G_αi/o_ are not involved in mediating the toxin’s primary activity.

In addition to the Gα subunits of heterotrimeric G proteins, ligand-bound GPCRs also activate the G_βγ_ complex, which acts on various effectors, including G protein-gated inwardly rectifying K+ channels, calcium channels, adenylyl cyclase, PLC, and phosphoinositide 3-kinase γ (PI_3_Kγ) [67]. While some of these actions were indirectly tested in the experiments described above, we decided to examine the effect on the toxin’s action of potent, selective, and cell-permeable PI_3_K inhibitors, LY294002 (reversible inhibitor [68,69]) and wortmannin (irreversible inhibitor [70,71]). However, neither inhibitor affected the LTX^N4C^-induced, LPHN1-mediated burst-like ACh exocytosis (Figure 6i and Figure S5j; Table 1).

Thus, the G_αq_/PLC pathway is strictly required to transduce the signal from LTX^N4C^-activated LPHN1 to generate bursts of ACh exocytosis. G_αs_/cAMP signaling (not necessarily induced by LPHN1 activation) is not essential for burst initiation but plays a modulatory role in shaping burst dynamics. By contrast, G_αi/o_ and G_βγ_/PI_3_K pathways are dispensable for these effects in the motor nerve terminal.

### 3.6. Store-Operated Ca^2+^ Entry Mediates the LPHN1-Induced Increase in Spontaneous Exocytosis

The preceding sections establish that LTX^N4C^-induced, LPHN1-mediated signaling elevates cytosolic Ca^2+^ via G_αq_-dependent PLC activation—a pathway classically associated with intracellular Ca^2+^ release and subsequent SOCE. We therefore investigated whether intracellular Ca^2+^ stores and SOCE underlie the toxin’s effects at the NMJ.

**Figure 7:**
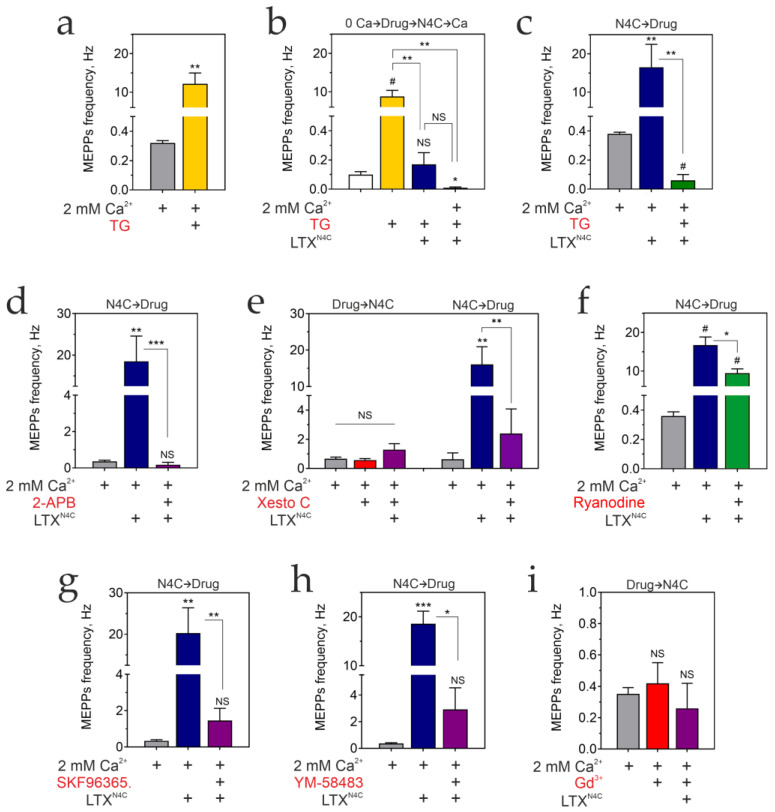
Ca^2+^ stores and SOCE are involved in the LTX-induced, LPHN1-mediated actions at the mouse NMJ. (**a**) MEPP frequencies in 2 mM Ca^2+^ and 10 μM TG; *n* = 4; *N* = 34. (**b**) MEPP frequencies in 0 Ca^2+^_e_ with or without 10 μM TG. 0.25 nM LTX^N4C^ was added after the end of TG-induced activity, followed by 2 mM Ca^2+^. Toxin failed to induce an increase in MEPP frequency; *n* = 3; *N* = 38. (**c**–**g**) Preparations were stimulated with 0.25 nM LTX^N4C^ in 2 mM Ca^2+^_e_. After the toxin’s action developed, treatment with the following inhibitors blocked or partially inhibited the LTX^N4C^ effect: (**c**) 10 μM TG; *n* = 4; *N* = 28; (**d**) 50 μM 2-APB; *n* = 3; *N* = 18; (**e**) 0.5 μM xestospongin C; *n* = 3; *N* = 9; (**f**) 100 μM ryanodine; *n* = 3; *N* = 15; (**g**) 50 μM SKF96365; *n* = 3; *N* = 25; (**h**) 100 μM YM58483; *n* = 3; *N* = 17. (**i**) Preparations incubated in 2 mM Ca^2+^ and 1 mM Gd^3+^ were stimulated with 0.25 nM LTX^N4C^, which failed to induce any MEPP frequency increase; *n* = 3; *N* = 19. Symbols next to bars indicate statistical significance compared to respective controls; other comparisons are shown by lines: *, *p* < 0.05; **, *p* < 0.01; ***, *p* < 0.001; #, *p* < 0.0001; NS, not significant.

To test the involvement of intracellular Ca^2+^ stores, we used the sarcoplasmic/endoplasmic reticulum Ca^2+^ ATPase (SERCA) pump inhibitor TG. In 2 mM Ca^2+^_e_, TG rapidly induced high-frequency bursts of MEPPs (Figure 7a) resembling those evoked by LTX^N4C^, though with slightly lower amplitude and shorter IBIs (Figure S6a). This activity persisted for >1 h. In Ca^2+^_e_-free solution, TG also triggered bursting (Figure 7b, yellow bar; Figure S6b), but the response subsided within ~10 min and resumed only upon re-addition of Ca^2+^_e_ (Figure S6b, left). This pattern suggests that TG depletes stores in discrete, spike-like events rather than via gradual leak, and that store refilling requires extracellular Ca^2+^.

Postsynaptically, TG increased MEPP amplitude and duration, consistent with our previous report [10]. Average MEPP amplitudes (determined within narrow V_m_ strata and normalized to −70 mV, as described in Section 2.3) were 0.82 ± 0.11 mV in Ca^2+^ buffer control and 2.5 ± 0.02 mV after TG addition (*n* = 3, *N* = 25 and 27 individual synapses, respectively; *p* < 0.001 Mann–Whitney U test).

The TG-induced Ca^2+^ store recycling could mask the effect of LTX^N4C^. Therefore, we added LTX^N4C^ after the store had been depleted in 0 Ca^2+^_e_ (Figure S6b, right). Subsequent addition of 2 mM Ca^2+^_e_ failed to restore synaptic activity (Figure 7b and Figure S6b, right; Table 1). Conversely, when TG was applied after LTX^N4C^ had already induced bursting in Ca^2+^_e_-containing solution, activity ceased immediately and did not resume (Figure 7c and Figure S6c; Table 1). Thus, both replete TG-sensitive stores and Ca^2+^_e_ are strictly required for LTX^N4C^-induced exocytosis.

Once it was established that the LPHN1-mediated effect in motor neurons depends on TG-sensitive stores, which respond to increases in IP_3_ levels, it was logical to test whether the toxin’s action in fact required the activation of IP_3_ receptors (IP_3_Rs). We tested 2-APB, which inhibits IP_3_ receptors [72,73] but at higher concentrations also blocks the ‘transient receptor potential canonical’ (TRPC), as well as TRPM channels [74]. At 50 µM—a concentration that preferentially targets IP_3_Rs—2-APB [72] rapidly suppressed LTX^N4C^-induced bursting to basal levels without affecting MEPP amplitude (Figure 7d and Figure S6d; Table 1). This indicates that IP_3_R activation is required for the LPHN1-mediated effect.

However, given the various known off-target effects of 2-APB [75,76], we decided to confirm that active IP_3_R are required for the massive LTX^N4C^-induced ACh release using another IP_3_R inhibitor, the non-competitive antagonist xestospongin C [77,78,79,80]. At 0.5 μM, a concentration that effectively blocks IP_3_Rs while minimizing reported off-target effects on VGCCs [81], xestospongin C strongly inhibited LTX^N4C^-induced ACh release, whether added before or after the toxin (Figure 7e and Figure S6e; Table 1). Although xestospongin C can exhibit side effects at higher concentrations, the close similarity of its inhibitory action to that of 2-APB—combined with the use of a concentration optimized for IP_3_R selectivity—supports the conclusion that IP_3_R activation likely mediates the LTX^N4C^-induced bursts of MEPPs.

In addition to IP_3_-sensitive stores, ryanodine-sensitive Ca^2+^ stores have been reported to reside in nerve terminals and contribute to neurotransmitter release [82]. Ryanodine, which at the concentrations used here blocks RyRs without affecting the IP_3_R [83,84,85], did not halt LTX^N4C^-induced bursting (Figure 7f; Table 1) but reduced overall MEPP frequency from 16.50 ± 7.07 Hz to 9.45 ± 2.59 Hz (*n* = 11 and 5, respectively), reflecting a ~40% decrease in burst magnitude. Thus, ryanodine-sensitive stores contribute partially to the LPHN1-mediated response, possibly as a secondary consequence of Ca^2+^ influx during SOCE.

Based on the critical role of intracellular Ca^2+^ stores in the effects of LTX^N4C^, we hypothesized that the toxin-evoked signal, via activation of LPHN1 and depletion of Ca^2+^ stores, stimulates SOCE. Loss of stored Ca^2+^ induces a conformational change in the store-resident protein ‘stromal interaction molecule’ (STIM1/2), which activates SOCCs on the plasma membrane, which may include Orai and/or TRPC-type channels. To examine whether SOCCs are involved in LPHN1-mediated LTX^N4C^ actions, we used several inhibitors of these channels.

The SOCC blockers SKF96365 (which inhibits TRPC channels and the STIM1–Orai1 interaction [86]) and YM58483 (a potent SOCE inhibitor [87,88]) both strongly suppressed and eventually abolished LTX^N4C^-induced bursting (Figure 7g,h and Figure S6f,g; Table 1).

We also tested the frequently used SOCE blocker Gd^3+^, which blocks Orai1–3 channels with high potency (nanomolar range) and inhibits multiple TRPC channels at micromolar concentrations [89,90]. Gd^3+^ inhibits SOCE with an *IC*_50_ of 18–28 nM [16]; we therefore used increasing Gd^3+^ concentrations to optimize SOCE blockade and assess its contribution to LTX^N4C^-induced effects. In the presence of 2 mM extracellular Ca^2+^, 20 nM Gd^3+^ produced approximately 67% inhibition of MEPP bursts (Table 1). Increasing the concentration to 100 μM Gd^3+^ resulted in a similar inhibition (71%), suggesting that while SOCE contributes to the LTX^N4C^ response, additional Ca^2+^ influx pathways independent of SOCCs are also involved. To block all Ca^2+^ entry, we applied 1 mM Gd^3+^, which is known to broadly inhibit plasma membrane Ca^2+^-ATPase and most other ion channels, effectively insulating the cell from Ca^2+^ influx and efflux [91]. Under this condition, LTX^N4C^-induced MEPP bursts were completely abolished, while basal transmission persisted (Figure 7i and Figure S6h; Table 1). These findings confirm that SOCE is required for the full LTX^N4C^ response and further demonstrate that the toxin’s action critically depends on Ca^2+^ influx, while without it, intracellular store mobilization alone is insufficient to trigger exocytosis.

Collectively, these experiments demonstrate that LTX^N4C^ activates LPHN1 to engage the canonical G_αq_-PLC-IP_3_ pathway, leading to depletion of TG-sensitive Ca^2+^ stores, opening of SOCCs, and Ca^2+^ influx. Ryanodine-sensitive stores contribute to this response, likely as a secondary amplification mechanism. However, the G_αq_-linked pathway cannot alone account for the toxin’s effects, because LTX^N4C^, unlike TG, does not cause any increase in spontaneous synaptic activity in the absence of Ca^2+^_e_. Thus, the toxin absolutely requires Ca^2+^ influx before it can release stored Ca^2+^. This absolute dependence on Ca^2+^ influx raised the possibility that LTX^N4C^ might also gate some Ca^2+^ channels—a hypothesis explored in the next section.

### 3.7. The Crucial Role of VGCCs

When searching for a channel that could mediate LTX^N4C^-induced Ca^2+^ influx, we relied on our earlier finding that in the neuroblastoma model system mentioned earlier [32], LTX^N4C^ action mediated by LPHN1 requires the activity of a Ca_V_2 channel. However, motor neurons and neuroblastoma cells have different repertoires of VGCCs [32], and different VGCC isoforms could be stimulated by LTX^N4C^ at the NMJ. Therefore, we investigated the role of several VGCC types in the LTX^N4C^ action using specific channel blockers.

**Figure 8:**
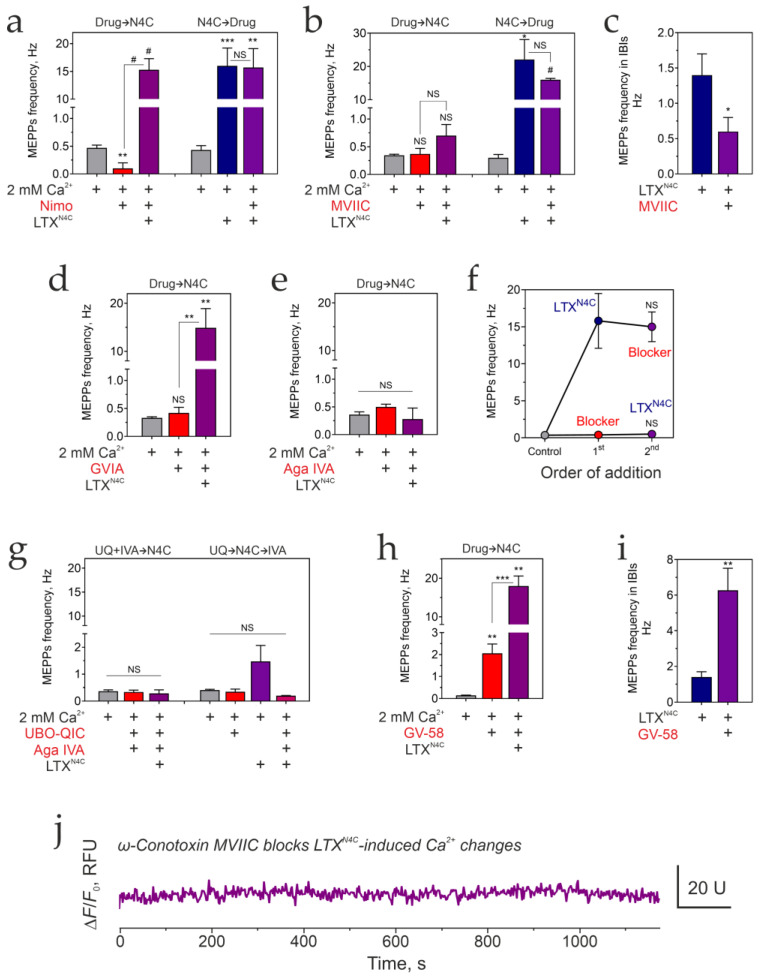
Ca_V_2.1 channels provide a trigger for LTX^N4C^-induced bursts of exocytosis, while Ca_V_1 channels modify their dynamics. Mouse neuromuscular preparations were incubated in 2 mM Ca^2+^ and stimulated by 0.25 nM LTX^N4C^. (**a**,**b**) Average MEPP frequencies induced by LTX^N4C^ before or after the application of Ca_v_ channel inhibitors: 10 μM nimodipine (*n* = 6; *N* = 32) to block Ca_V_1 channels (**a**), or 1 μM ω-conotoxin MVIIC (*n* = 4; *N* = 36) to block Ca_V_2.1/2.2 channels (**b**). (**c**) ω-Conotoxin MVIIC significantly reduces MEPP frequencies within the IBIs of LTX^N4C^-induced bursts; *n* = 5. (**d**,**e**) Average MEPP frequencies induced by LTX^N4C^ after the application of more selective Ca_V_2 channel inhibitors: 50 nM ω-conotoxin GVIA (*n* = 4; *N* = 32) to block Ca_V_2.2 channels (**d**), or 200 nM ω-agatoxin IVA (*n* = 6; *N* = 31) to block Ca_V_2.1 channels (**e**). (**f**) Ca_V_2.1 blockers only affect LTX^N4C^ actions if added before the toxin, but fail to do so when added after LTX^N4C^; *n* = 10; *N* = 67. (**g**) ω-Agatoxin IVA (200 nM) blocks all LTX^N4C^-induced increase in MEPP frequency when added together with or after the G_αq_ blocker UBO-QIC; *n* = 4; *N* = 32. (**h**,**i**) The Ca_V_2 agonist GV-58 (50 μM) increases the basal spontaneous MEPP frequency (**h**), but does not affect the average MEPP frequency induced by LTX^N4C^; *n* = 3; *N* = 36. However, GV-58 significantly increases MEPP frequency within IBIs (**i**); *n* = 3; *N* = 36. Symbols next to bars indicate statistical significance compared to respective controls; other comparisons are shown by lines: *, *p* < 0.05; **, *p* < 0.01; ***, *p* < 0.001; #, *p* < 0.0001; NS, not significant. (**j**) LTX^N4C^ fails to induce any changes in Ca^2+^_cyt_ in nerve terminals pretreated with ω-conotoxin MVIIC. Muscle preparations were preloaded with Fluo-4-AM (see Figure 2) and treated with 1 μM ω-conotoxin MVIIC, followed by the addition of 0.25 nM LTX^N4C^ and recording of intracellular fluorescence.

First, we used nimodipine, a specific blocker of Ca_V_1 (L-type) VGCCs [92]. When added before LTX^N4C^, it significantly decreased the basal MEPP frequency. It failed to abolish the toxin-induced bursts of release (Figure 8a and Figure S7a; Table 1) but profoundly altered their dynamics. In nimodipine, the bursts themselves were significantly smaller, with the intra-burst MEPP frequency decreasing from 36.5 ± 9.0 Hz to 22.3 ± 3.24 Hz (*n* = 3, *N* = 12).

Strikingly, this reduction in burst size was counterbalanced by an increase in burst frequency, such that the overall average MEPP frequency remained unchanged. In a reverse experiment, when nimodipine was added after LTX^N4C^, the previously developed toxin-induced bursts continued unchanged, but then ceased altogether after 10–15 min (Figure 8a and Figure S7a; Table 1). This effect resembled the cessation of TG-induced MEPP bursts in the absence of Ca^2+^e (Figure S6b), when Ca^2+^ stores were depleted and could not be replenished. We interpret the nimodipine-altered burst pattern—smaller but more frequent bursts—and their delayed cessation as the result of LTX^N4C^-induced store depletion combined with a failure to refill them when Ca_V_1 channels are blocked by nimodipine. In addition, nimodipine slightly increased MEPP amplitudes (Figure S7b), indicating also a postsynaptic effect. Overall, our findings suggest that Ca_V_1 channels are not the primary trigger for LTX^N4C^-induced bursts but instead play a critical role in refilling intracellular Ca^2+^ stores, thereby modulating the pattern of spontaneous release at the mouse NMJ.

ω-Conotoxin MVIIC, a broad-spectrum blocker of Ca_V_2 family VGCCs (N- and P/Q-type) [93], abolished the effect of LTX^N4C^ when added before the toxin (Figure 8b and Figure S7c, left; Table 1). Strikingly, when MVIIC was applied after LTX^N4C^, it only slightly inhibited the average MEPP frequency but failed to affect the bursts themselves (Figure 8b and Figure S7c, right; Table 1). Although the inhibition of average LTX^N4C^ activity was statistically insignificant, we noticed that MVIIC significantly decreased MEPP frequency within IBIs (Figure 8c). This indicates that Ca^2+^ influx through Ca_V_2 channels is important before the start of LTX^N4C^-induced bursting and continues to contribute to background MEPP frequency after bursts begin (see also below).

To differentiate between the roles of Ca_V_2.1 (P/Q-type) and Ca_V_2.2 (N-type) VGCCs, we used selective blockers: ω-agatoxin IVA (for Ca_V_2.1) [94] and ω-conotoxin GVIA (for Ca_V_2.2) [95], respectively. GVIA failed to cause any significant change in LTX^N4C^ activity (Figure 8d and Figure S7d; Table 1). By contrast, IVA—like MVIIC—abolished the toxin’s effect when added before LTX^N4C^ (Figure 8e and Figure S7e; Table 1). We concluded that among all VGCCs tested, only Ca_V_2.1 is involved in the initiation of LTX^N4C^ action.

Thus, the efficiency of MVIIC and IVA in blocking the LTX^N4C^ action depends critically on the order of addition (Figure 8f). If a channel blocker is applied first, subsequent LTX^N4C^ application is ineffective. If LTX^N4C^ is allowed to act first, the blocker fails to inhibit the established activity.

These findings led us to propose that LTX^N4C^ acts through two complementary pathways: G_αq_-mediated priming of the Ca^2+^ store and low-level Ca^2+^ influx through Ca_V_2.1. Without Ca^2+^ influx, the Gq pathway alone is insufficient to mobilize stores and initiate SOCE. However, once Ca^2+^ influx occurs and SOCE develops, the response becomes self-sustaining and the initiating Ca_V_2.1 channels are no longer required. In this model, Ca^2+^ entering through Ca_V_2.1 provides the initial Ca^2+^cyt signal that synergizes with the primed G_αq_ pathway to efficiently mobilize Ca^2+^ stores. Indeed, as shown above, inhibition of G_αq_ drastically reduces—but does not completely block—the LTX^N4C^ effect. This residual activity likely reflects a second, parallel mechanism LTX^N4C^: direct or indirect activation of Ca_V_2.1 channels, leading to slow Ca^2+^ entry and a moderate increase in the frequency of spontaneous exocytosis (and consequently MEPP frequency).

We tested this hypothesis by applying IVA together with or after UBO-QIC. Co-application of ω-agatoxin IVA and UBO-QIC completely abolished all LTX^N4C^-induced increases in synaptic activity (Figure 8g and Figure S7f; Table 1). Furthermore, the residual LTX^N4C^-induced activity remaining after UBO-QIC inhibition was completely blocked by subsequent addition of IVA (Figure 8g and Figure S7f; Table 1).

Our conclusion about the complementarity of G_αq_ and Ca_V_2.1 actions was further tested using GV-58, an agonist of Ca_V_2 family VGCCs [96]. When added before LTX^N4C^, GV-58 significantly increased the basal MEPP frequency (Figure 8h and Figure S7g,h; Table 1). GV-58 did not substantially enhance the peak activity of LTX^N4C^ compared to the toxin alone, but very strongly augmented the average MEPP frequency during IBIs (Figure 8i and Figure S7h). This result corroborates our hypothesis that Ca_V_2.1 activation not only acts as an additional trigger for Ca^2+^ mobilization but also sustains an elevated level of Ca^2+^cyt that is not strictly required once bursting develops but may be important for persistent bursting activity.

The strong inhibition of LTX^N4C^-induced MEPP bursts by Ca_V_2.1 blockers prompted us to investigate whether these agents also prevent the toxin-stimulated intracellular Ca^2+^ spikes and waves (Figure 2). In neuromuscular preparations loaded with Fluo-4, pretreatment with ω-conotoxin MVIIC eliminated the typical cytosolic Ca^2+^ increases normally triggered by LTX^N4C^ (Figure 8j and Figure S7i). Combined with the failure of LTX^N4C^ to generate any Fluo-4 signal in the absence of Ca^2+^_e_ (Figure 2h), this observation confirms that the dynamic Ca^2+^ changes depend on LTX^N4C^-evoked Ca^2+^ influx across the plasma membrane. Along with the finding that IP_3_R inhibition blocks LTX^N4C^-induced MEPP bursts (Figure 7), these results suggest that LTX^N4C^-triggered burst-like exocytosis requires both Ca^2+^ entry through Ca_V_2.1 channels and IP_3_R-mediated store mobilization.

Finally, to exclude the possibility that LTX^N4C^ acts by depolarizing the membrane of motor terminals, we applied TTX, a highly specific blocker of voltage-gated Na^+^ channels (VGSCs) [97,98]. Tested across various experimental conditions, TTX had no effect on basal MEPP frequency, burst frequency, burst duration, burst magnitude, or IBIs (Table 1). These findings indicate that LTX^N4C^ does not act by inducing VGSC-dependent depolarization of the presynaptic or axonal membrane.

Taken together, these results reveal a novel mechanism through which GPCRs, in particular LPHN1, can modulate spontaneous exocytosis by depleting intracellular Ca^2+^ stores and activating SOCE.

## 4. Discussion

The present study establishes a comprehensive mechanism by which LTX^N4C^, acting through the presynaptic GPCR LPHN1, induces burst-like spontaneous ACh release at the vertebrate NMJ. Our findings reveal a multi-step signaling cascade that begins with toxin binding to LPHN1 and culminates in SOCE, with VGCCs playing distinct and unexpectedly dissociable roles in initiating versus sustaining this response.

### 4.1. LPHN1 as a Master Regulator of Spontaneous Quantal Release

A central conclusion from our work is that LPHN1 serves as a dedicated receptor for LTX^N4C^ at the motor nerve terminal, and its activation is both necessary and sufficient to generate bursts of quantal ACh release. Several lines of evidence support this assertion. First, LPHN1 KO nearly abolishes LTX^N4C^-induced bursting, reducing it to rare, low-frequency events (Figure 3). Second, the recombinant anti-LPHN1 scFv antibody A1, which competes with αLTX for the same binding site on the NTF, recapitulates the toxin’s effect—inducing bursts that are morphologically similar to those evoked by LTX^N4C^, albeit with lower frequency (Figure 3h–j). Third, LPHN1 is unequivocally localized to the presynaptic membrane at both frog and mouse NMJs (Figure 4 and Figure 5), with its distribution precisely matching active zone organization in frog preparations. This presynaptic localization, combined with the denervation experiments showing LPHN1 is axonally transported (Figure 4c), establishes LPHN1 as a motor neuron-derived protein positioned to directly regulate neurotransmitter release. Fourth, the spatial pattern of LTX^N4C^ binding to mouse motor terminals precisely matches that of LPHN1 immunostaining (Figure 5e,g).

The residual activity in LPHN1 KO preparations, together with persistent LTX^N4C^ binding at KO NMJs (Figure 5g,h), implies a secondary, low-affinity receptor—perhaps LPHN3, PTPσ, or Ca_V_2.1 channels themselves—that can weakly engage the same downstream machinery. This redundancy may ensure some level of signaling robustness, but the quantitative dominance of LPHN1 is unmistakable.

### 4.2. The G_αq_-PLC-IP_3_ Axis Is Required for Burst Generation

Our pharmacological dissection reveals that LPHN1 signals through the canonical G_αq/11_ pathway to generate bursts. The G_αq_ inhibitor UBO-QIC completely prevents bursting when added before LTX^N4C^ and rapidly halts ongoing bursts (Figure 6a). Similarly, the PLC inhibitor U73122 blocks burst initiation and terminates established activity, while its inactive analog U73343 has no effect (Figure 6b,c). These results firmly establish that G_αq_ and PLC are not merely modulatory bystanders but absolutely required relay links for the burst phenotype.

Remarkably, however, neither UBO-QIC nor U73122 fully suppresses the elevated MEPP frequency observed during IBIs (Figure 6a,b). This dissociation between burst generation and IBI maintenance suggests that two parallel mechanisms operate downstream of LPHN1: one G_αq_-dependent pathway that produces high-frequency bursts, and a second, G_αq_-independent pathway that sustains moderate release during IBIs. The identity of this second pathway remained unclear until our VGCC experiments (discussed below) proved it involves Ca_V_2.1 activation.

### 4.3. Store-Operated Ca^2+^ Entry as the Central Mechanism

The involvement of G_αq_ and PLC immediately suggested a role for IP_3_-mediated Ca^2+^ store release. Indeed, multiple lines of evidence converge on SOCE as the central mechanism driving LTX^N4C^-induced exocytosis. First, TG, which depletes stores by inhibiting SERCA pumps, recapitulates the burst phenotype (Figure 7a). Second, LTX^N4C^ fails to induce activity when stores are pre-depleted by TG in Ca^2+^-free conditions (Figure 7b), and conversely, TG (applied after LTX^N4C^) terminates ongoing bursts (Figure 7c). Third, the IP_3_R inhibitor 2-APB rapidly suppresses LTX^N4C^-induced bursting (Figure 7f). Fourth, and most definitively, multiple SOCE inhibitors—SKF96365, YM58483, and Gd^3+^—all abolish the toxin’s effect (Figure 7e,g,h).

These findings collectively demonstrate that LTX^N4C^, via LPHN1 and G_αq_-PLC-IP_3_, depletes intracellular Ca^2+^ stores, which in turn activate SOCCs to drive sustained Ca^2+^ influx and exocytosis. The striking similarity between LTX^N4C^-induced Ca^2+^cyt fluctuations (Figure 2f) and MEPP frequency bursts (Figure 1a) strongly suggests that the periodic nature of release reflects underlying oscillations in store depletion and refilling—a phenomenon well-documented in other cell types [99,100].

Interestingly, ryanodine-sensitive stores also contribute, as ryanodine reduces intraburst frequency by ~40% (Figure 7e). This likely represents CICR amplification, where Ca^2+^ entering through SOCCs or Ca_V_2.1 channels triggers additional release via RyRs, creating a positive feedback loop that sharpens burst dynamics. Such CICR mechanisms are well-established in neurons [23,101,102,103,104] and may explain why bursts are so sharply defined rather than gradual elevations in release.

The two types of LTX^N4C^-induced bursts (termed here spikes and waves), observed both electrophysiologically (Section 3.1) and in presynaptic Ca^2+^cyt fluorescence (Section 3.2), suggest that the SOCCs themselves might operate in different functional states. Our data point to a modulatory role for cAMP in this switch. Inhibition of adenylyl cyclase by ddAdo or SQ22536 strongly increased the duration of MEPP bursts induced by LTX^N4C^ and converted many spikes to longer waves (Figure 6 and Figure S5). This indicates that cAMP-dependent phosphorylation could normally promote early burst termination, resulting mainly in short spikes. This modulation could occur through direct effects on SOCC inactivation kinetics or on other elements of the release machinery. The source of this cAMP signal remains unclear, but it is unlikely to be mediated by G_αi/o_, as PTX failed to modify LTX^N4C^ effects (Figure 6 and Figure S5).

### 4.4. The Surprising Dual Role of Voltage-Gated Ca^2+^ Channels

Our VGCC experiments yielded perhaps the most unexpected and mechanistically illuminating results. Ca_V_2.1 (P/Q-type) channels are clearly required for burst initiation: ω-agatoxin IVA or ω-conotoxin MVIIC completely prevents LTX^N4C^ effects when added before the toxin (Figure 8b,e). However, once bursting is established, these same blockers fail to inhibit ongoing activity (Figure 8b,f). This order-dependent effect reveals that Ca_V_2.1 channels serve as an initiation trigger but are not absolutely required for maintenance—a striking dissociation that distinguishes initiation from perpetuation mechanisms.

We interpret this as follows: LTX^N4C^ binding to LPHN1 primes the G_αq_ pathway but cannot efficiently mobilize stores without an initial Ca^2+^ “spark”. This finding is fully supported by previous observations clearly demonstrating a poor ability of increased cytosolic IP_3_ to stimulate the opening of IP_3_R channels without a concomitant increase in Ca^2+^_cyt_ concentration [105]. This spark comes from Ca_V_2.1 channels, which may be activated either directly by the toxin itself or indirectly through G_α_ signaling. For example, diacylglycerol produced by activated G_αq_ could modulate Ca_V_2.1 channel open probability directly or via protein kinase C-dependent phosphorylation [106,107]. The distinct delay before the onset of toxin activity (Figure S1f) implies a multi-step signaling process and argues against a direct LTX^N4C^-Ca_V_2.1 interaction. Once this initial Ca^2+^ enters, it synergizes with IP_3_ to trigger store release, which then activates SOCE. The resulting Ca^2+^ influx through SOCCs sustains the response autonomously, rendering the initiating Ca_V_2.1 channels dispensable. This model elegantly explains why Ca_V_2.1 blockers work only when applied first: they prevent the initial spark, but once the SOCE engine is running, blocking the spark has no effect.

The residual IBI activity that persists after G_αq_ inhibition by UBO-QIC (Figure 6a) is completely eliminated by ω-agatoxin IVA (Figure 8g), suggesting that Ca_V_2.1 channels also provide a parallel, G_αq_-independent pathway that sustains a moderate release rate between bursts. This could represent direct toxin action on the channels themselves or the activation of another receptor, such as LPHN3 or PTPσ. Although LPHN3 is present in motor nerve terminals, its level (Figure 4) and affinity for LTX^N4C^ [48] are too low to justify the high amount of bound toxin in the absence of LPHN1 (~30% of binding to WT NMJs). Furthermore, the non-LPHN1 toxin binding in LPHN1 KO NMJs is tightly localized to central areas of the presynaptic membrane (Figure 5), a distribution that intriguingly aligns with the known clustering of Ca^2+^ channels at active zones. This spatial coincidence raises the possibility of a direct, albeit lower-affinity, interaction between LTX^N4C^ and the channels themselves, a hypothesis that warrants further investigation.

Ca_V_1 (L-type) channels play an entirely different role. Nimodipine does not prevent burst initiation but alters burst dynamics—making bursts smaller but more frequent—and causes delayed cessation when added after LTX^N4C^ (Figure 8a). This pattern closely resembles TG-induced burst cessation in Ca^2+^-free conditions (Figure S6b), leading us to propose that Ca_V_1 channels are required for store refilling. In this model, Ca^2+^ entering through L-type channels during or between bouts of SOCE replenishes stores, allowing repeated cycles of depletion and release. When these channels are blocked, stores progressively empty and bursts eventually cease—explaining the delayed cessation in the “nimodipine after” experiment.

### 4.5. A Unified Model

**Figure 9:**
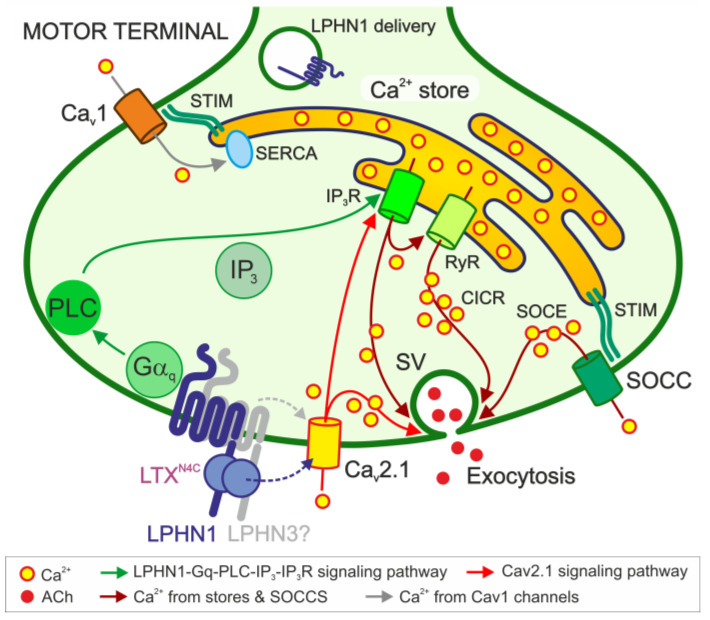
Schematic model of LTX^N4C^-induced, LPHN1-mediated ACh release at the NMJ. Arrows showing the two main signaling pathways of LTX^N4C^ and Ca^2+^ actions are defined in the legend. Dotted lines indicate hypothetical pathways of Ca_V_2.1 activation by LTX^N4C^. See text for details.

Synthesizing these findings, we propose the following model for LTX^N4C^ action at the NMJ (Figure 9):

- Binding and priming: LTX^N4C^ binds the NTF of presynaptic LPHN1, inducing a conformational change that activates G_αq_.
- Initial Ca^2+^ spark: In parallel, but after a distinct delay, which implies a multi-step signaling process, LTX^N4C^ promotes Ca^2+^ influx through Ca_V_2.1 channels. This mechanism remains unclear.
- Store depletion: The combination of IP_3_ (from G_αq_-PLC activity) and the initial Ca^2+^ spark triggers IP_3_R-mediated Ca^2+^ release from TG-sensitive stores. This release may be amplified by CICR via RyRs.
- SOCE activation: Store depletion activates SOCCs (likely Orai1–3 channels, as motor neurons only express very low levels of TRPC1, 3, and 6 [32]), producing sustained Ca^2+^ influx that drives high-frequency exocytosis during bursts. Massive CICR via RyRs could contribute to some or all bursts.
- Store refilling: During and between bursts, Ca^2+^ entering through Ca_V_1 channels refills stores, enabling repeated cycles of depletion and release.
- Modulation: cAMP signaling (via G_αs_ activated by LPHN1 or another GPCR) regulates burst duration, actively promoting burst termination (Figure 6f).

This model accounts for all our key observations: the absolute requirement for Ca^2+^ influx (Figure 1e,f), the periodic nature of release (Figure 1 and Figure 2), the necessity of G_αq_-PLC-IP_3_ signaling (Figure 6 and Figure 7), the order-dependent effects of Ca_V_2.1 blockers (Figure 8), and the modulatory roles of Ca_V_1 and cAMP (Figure 6f and Figure 8a).

### 4.6. Broader Implications

Our findings have implications beyond toxin mechanism. They reveal that a GPCR can drive sustained neurotransmitter release through SOCE—a pathway more commonly associated with non-excitable cells [16,108]. This suggests that SOCE combined with CICR may be a general mechanism for modulating presynaptic activity, particularly during prolonged stimulation or under pathophysiological conditions where sustained release is required.

The dissociation between burst initiation (Ca_V_2.1-dependent) and maintenance (SOCE-dependent) provides a new framework for thinking about presynaptic Ca^2+^ signaling. It demonstrates that different Ca^2+^ sources can serve distinct temporal roles, with VGCCs providing rapid, local signals and SOCE supplying sustained, global Ca^2+^ elevations. This division of labor may be a general principle at synapses.

Finally, the somewhat unexpected role of Ca_V_1 channels in store refilling suggests a functional coupling between Ca_V_1 channels and intracellular stores. Similar observations were reported previously in central neurons: Ca_V_1 channels play a specialized role in maintaining intracellular Ca^2+^ homeostasis in the soma and dendrites by refilling IP_3_-sensitive stores during subthreshold electrical activity [109,110], while the channel’s activity is regulated by its interaction with STIM1 [111]. Presynaptic Ca_V_1 channels are known to play an important role in pathological conditions like Alzheimer’s and Parkinson’s diseases, where they mediate elevated Ca^2+^ influx [112]. However, a clear physiological role for Ca_V_1 at mammalian motor nerve terminals has not been previously appreciated. Our findings suggest that this refilling function may represent a fundamental mechanism, linking acute Ca^2+^ signaling to the maintenance of synaptic efficacy and potentially to longer-term forms of presynaptic plasticity.

Bursts of MEPP frequency can also be induced under various pathophysiological conditions. For example, when ACh esterase (AChE) is inhibited by anticholinesterases such as neostigmine, carbamate, or organophosphorus compounds, nerve stimulation evokes exaggerated responses—including spastic hypercontractions of muscle and massive bursts of MEPPs. Similarly, a marked post-tetanic increase in MEPP frequency has been reported in paralyzed muscles undergoing reinnervation by inactive motor axons [113]. In both cases, AChE activity is reduced—either pharmacologically or as a consequence of chronic inactivity—suggesting that unhydrolyzed ACh may act via positive feedback on presynaptic terminals. Given that LPHNs are conserved across species and enriched at synapses, they are likely to serve important physiological functions in motor terminals. One intriguing possibility is that they could participate in the feedback regulation of neurotransmitter release, for instance, by modulating the activity of voltage-gated calcium channels (Ca_V_2.x) in response to changes in synaptic activity, such as those triggered by ACh accumulation. If so, the MEPP bursts observed upon LTX^N4C^-induced LPHN1 activation could reflect a transient, non-physiological engagement of this same regulatory pathway.

### 4.7. Limitations and Future Directions

One potential limitation of this study is that many of the pharmacological agents used are not absolutely specific and could exhibit off-target effects. To address this, we employed convergent pharmacology and genetics, such that our conclusion—that LTX^N4C^ activates the LPHN1–G_αq_–PLC–IP_3_–IP_3_R–Ca^2+^ axis—rests on the cumulative weight of six independent manipulations. All key nodes of this pathway were tested by either genetic or pharmacological interventions. While each agent carries a risk of off-target activity, the probability that six mechanistically distinct interventions would coincidentally converge on the same pathway due to spurious effects is extremely low (*p* = 0.000729 assuming 30% off-target probability per agent, or 0.000064 at 20%).

However, several questions remain. The molecular identity of the SOCCs mediating LTX^N4C^ effects requires clarification. All three Orai channels are abundant in motor neurons, but while the expression of TRPC1, 3, and 6 channels is very low [32], their subcellular distribution is unknown, and they could potentially contribute to LTX^N4C^ effects. The inhibitors we used could not distinguish between Orai and TRPC channels. The roles of STIM1/2 could be studied by KO or knockdown experiments. STIM2 knockdown in LPHN1-expressing neuroblastoma cells did not change the response to LTX^N4C^, but STIM2 is much more prevalent in motor neurons and might play a more significant role. The mechanism by which LTX^N4C^ initially activates Ca_V_2.1 channels also remains unclear; direct electrophysiological recording from motor nerve terminals, though technically challenging, could resolve whether the toxin modulates channel gating.

The residual activity in LPHN1 KO preparations, while minimal, hints at an auxiliary receptor. Identifying this molecule—perhaps LPHN3 or PTPσ—could reveal whether LPHN1 is part of a larger signaling complex at active zones. Finally, the cAMP-mediated burst termination mechanism (Figure 6f) deserves exploration: does this represent a negative feedback loop, and what GPCR activates it?

In conclusion, our work establishes LPHN1 as a presynaptic GPCR that drives burst-like ACh release through a novel mechanism combining G_αq_-PLC signaling, Ca_V_2.1-triggered store depletion, and SOCE. This pathway reveals unexpected complexity in the regulation of spontaneous neurotransmitter release and may represent a general mechanism for GPCR-mediated presynaptic modulation.

## 5. Conclusions

We show that LPHN1 is presynaptic at the mouse NMJ and is the main mediator of LTX^N4C^ action. The toxin induces the LPHN1-G_αq_-PLC-IP_3_ signaling cascade, which primes IP_3_R on Ca^2+^ stores. LTX^N4C^ also activates Ca_V_2.1 channels, which provide Ca^2+^_cyt_. A synergistic action of IP_3_ signaling and Ca^2+^_cyt_ triggers Ca^2+^ store mobilization and oscillation of presynaptic Ca^2+^_cyt_. Ca^2+^_cyt_ spikes cause bursts of ACh exocytosis. Thus, LTX^N4C^ hijacks a physiological mechanism that is likely to contribute significantly to the regulation of synaptic activity under normal conditions.

## Figures and Tables

**Table 1:** The inhibitory effects of different compounds on LTX^N4C^-induced increase in MEPP frequency at the mouse NMJ.

Compound	Concentration, μM	Inhibition, % ^1^	Significance	Inhibition, % ^2^	Significance
BAPTA-AM	500	99.95 ± 3.40	**		
EGTA-AM	1000	95.22 ± 4.92	**		
UBO-QIC	1	90.05 ± 7.82	**	86.38 ± 8.03	*
U73122	10	90.89 ± 7.07	**	85.20 ± 4.54	**
U73343	10	−6.87 ± 9.42	NS	−8.96 ± 9.42	NS
ddAdo	100	−4.52 ± 6.14	NS		
SQ22536	10	1.25 ± 4.10	NS		
	25	17.84 ± 2.24	NS		
PTX	0.001	−6.41 ± 2.19	NS		
	0.02	1.82 ± 4.31	NS		
CTX	0.005	1.57 ± 3.28	NS		
LY294002	30	−1.82 ± 6.21	NS		
Wortmannin	1	10.90 ± 7.14	NS		
TG	10	99.57 ± 6.00	***	101.99 ± 1.51	**
2-APB	50			100.99 ± 6.07	***
Xestospongin C	5	95.91 ± 4.87	***	88.59 ± 8.77	**
Ryanodine	100			44.37 ± 7.50	*
SKF96356	50			94.38 ± 3.09	**
YM-58483	100			85.96 ± 8.03	*
Gd^3+^	0.02	67.01 ± 4.92	*		
	100	71.54 ± 3.52	*		
	1000	100.55 ± 0.36	***		
Nimodipine	10	4.51 ± 3.77	NS	1.93 ± 4.67	NS
ω-Conotoxin MVIIC ^3^	1	98.37 ± 6.00 ^3^	**	27.98 ± 6.01 ^3^	NS
ω-Conotoxin GVIA	0.05	12.60 ± 5.66	NS		
ω-Agatoxin IVA	0.2	100.51 ± 3.01	***		
UBO-QIC+Agatoxin IVA	1 + 0.2	100.48 ± 4.00	***	101.27 ± 2.50	***
GV-58	50	−5.75 ± 3.29	NS		
Tetrodotoxin (TTX)	1	1.52 ± 5.41	NS		

^1^ Order of addition: Compound → LTX^N4C^. ^2^ Order of addition: LTX^N4C^ → Compound. ^3^ The effect of ω-conotoxin MVIIC differs depending on the order of addition. *, *p* < 0.05; **, *p* < 0.01; ***, *p* < 0.001; NS, not significant.

## Data Availability

The original contributions presented in this study are included in the article/Supplementary Material. Further inquiries can be directed to the corresponding author.

## Supplementary Materials

The following supporting information can be downloaded at: https://www.mdpi.com/article/10.3390/cells15090821/s1, Figure S1: Characteristics of LTX^N4C^-induced spontaneous quantal neurotransmission at the mouse NMJ. Figure S2: Nerve terminal-specific Ca^2+^_cyt_ signals can be resolved even when mouse muscle fibers are unintentionally loaded with Fluo-4. Figure S3: The scFv antibody A1 interacts with LPHN1 rather than αLTX. Figure S4: LPHN1 in the frog. Figure S5: G_αq_ signaling is required for LTX^N4C^-induced, LPHN1-mediated bursts of exocytosis. Figure S6: Ca^2+^ stores and SOCE are involved in the LTX-induced, LPHN1-mediated actions at the mouse NMJ. Figure S7: Ca_V_2.1 channels provide a trigger for LTX^N4C^-induced bursts of quantal release, while Ca_V_1.x channels modify their dynamics.

## Abbreviations

The following abbreviations are used in this manuscript:

Abbreviation	Explanation
ACh	Acetylcholine
AChE	Acetylcholinesterase
AChR	Acetylcholine receptor
ADGRL1	Adhesion G-protein-coupled receptor L-type 1
AM	Acetoxymethyl ester
Ca^2+^_cyt_	Cytosolic Ca^2+^
Ca^2+^_e_	Extracellular Ca^2+^
Ca_v_	α-Subunit of a VGCC
CICR	Ca^2+^-induced Ca^2+^ release
CTF	C-terminal fragment
CTX	Cholera toxin
FDB	Flexor digitorum brevis (muscle)
GPCR	G-protein-coupled receptor
GST	Glutathione-S-transferase
HRP	Horseradish peroxidase
IBIs	Inter-burst intervals
IP_3_	Inositol 1,4,5-trisphosphate
IP_3_R	IP_3_ receptor
KO	Knockout
LPHN	Latrophilin (1–3)
LTX^N4C^	Mutant α-latrotoxin, which is unable to form membrane pores
mAb	Monoclonal antibody
MEPPs	Miniature end-plate potentials
mf	Muscle fiber
NMJ	Neuro-muscular junction
NRXN1α	Neurexin Iα
NTF	N-terminal fragment
PBS	Phosphate-buffered saline
PI_3_K	Phosphoinositide 3-kinase
PLC	Phospholipase C
PTPσ	Protein tyrosine phosphatase σ
PTX	Pertussis toxin
ROI	Region of interest
RT-qPCR	Reverse-transcription quantitative PCR
RyR	Ryanodine receptor, Ca^2+^ release channel
scFv	Single-chain variable fragment (antibody)
SCVH	Spinal cord ventral horn
SDS	Sodium dodecyl sulfate
SERCA	Sarcoplasmic/endoplasmic reticulum Ca^2+^-ATPase (Ca^2+^ pump)
SOCC	Store-operated Ca^2+^ channel
SOCE	Store-operated Ca^2+^ entry
STIM1/2	Stromal interaction molecules 1/2
SV	Synaptic vesicle
TG	Thapsigargin
TRPC	Transient receptor potential canonical
TTX	Tetrodotoxin
VGCC	Voltage-gated Ca^2+^ channel
VGSC	Voltage-gated Na^+^ channels
V_m_	Resting membrane potential
WT	Wild-type
αBuTX	α-Bungarotoxin
αLTX	α-Latrotoxin
