# Supplementary material for: Latrophilin-1-Mediated Gαq Signaling, Store-Operated Ca2+ Entry, and CaV2.1 Activation Control Spontaneous Exocytosis at the Mouse Neuromuscular Junction
Source: Cells. 2026 Apr 30;15(9):821. doi: 10.3390/cells15090821 (PMC13162755; doi:10.3390/cells15090821)
Supplement: Supplementary file 1 [file cells-15-00821-s001.zip › cells-4198661-supplementary.pdf]

cells

MDPI

Supplementary material

Latrophilin-1-mediated $G_{\alpha q}$ signaling, store-operated Ca^{2+} entry, and $Ca_V2.1$ activation control spontaneous exocytosis at the mouse neuromuscular junction

Evelina Petitto[1,†], Frédéric A. Meunier[2,‡], Sara Fidalgo[2,§], Cesare Colasante[3,‖], Jennifer K. Blackburn[1,¶], Richard R. Ribchester[4], Yuri A. Ushkaryov[1,2*]

3. Results

3.1. LTX^{N4C} causes bursts of high-frequency neurotransmitter release at the mouse NMJ

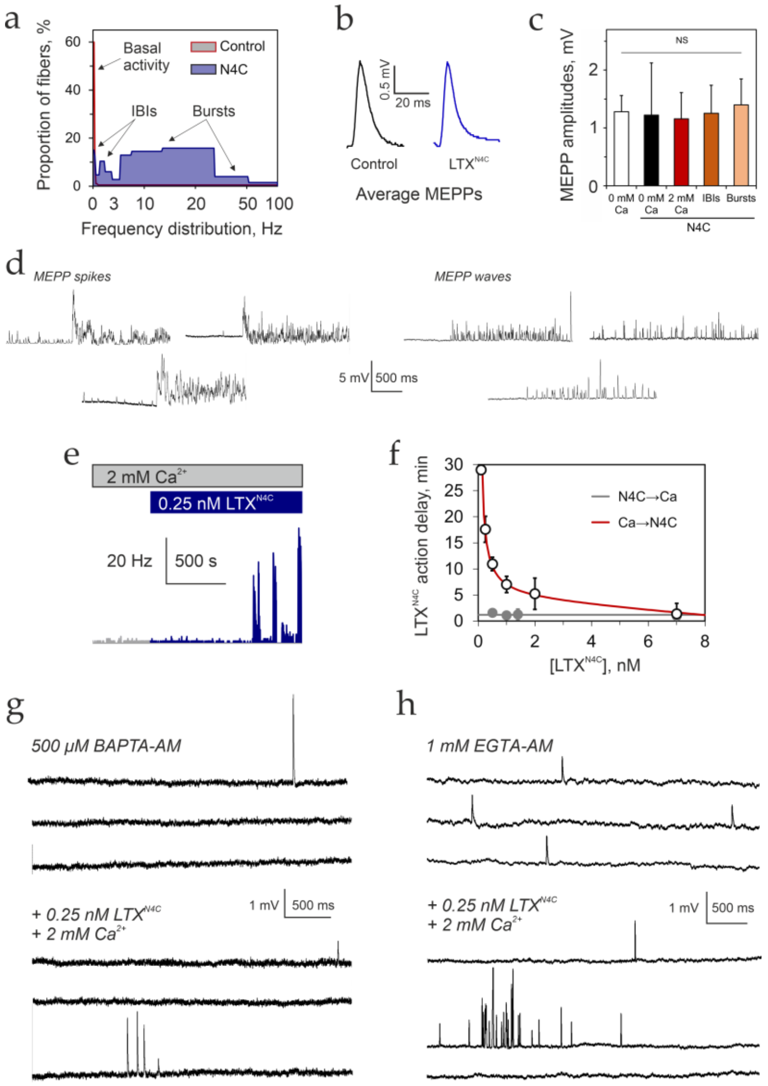

Figure S1. Characteristics of LTX^{N4C}-induced synaptic transmission at the mouse NMJ. (**a**) Statistical distribution of MEPP frequencies at NMJs bathed in 2 mM Ca^{2+}_e in the absence (control) or presence (^{N4C}) of 0.25 nM LTX^{N4C}. (**b**) Average MEPP waveforms under basal conditions (2 mM Ca^{2+}; control) and after the onset of LTX^{N4C} action; n = 500 events. (**c**) Quantification of MEPP amplitudes under control conditions and after LTX^{N4C} application. Statistical significance: NS, non-significant; n = 12.

(d) Representative MEPP spikes and MEPP waves, which exhibit different on-rates and mean frequencies. (**e**) Time course of MEPP frequency following application of 0.25 nM LTX^{N4C} in the presence of 2 mM Ca^{2+}_{e}. Note the delay before the onset of toxin-induced activity. (**f**) Dose-response curves for the onset of synaptic activity induced by LTX^{N4C}, comparing two experimental protocols: toxin added before (gray) or after Ca^{2+}_{e} (red). The curves are significantly different at $[LTX^{N4C}] \leq 2$ nM ($p < 0.001$; FANOVA). (g, h) V_m recordings from NMJs preloaded with intracellular BAPTA or EGTA; MEPPs appear as upward deflections.

3.2. LTX^{N4C} increases cytosolic Ca^{2+} levels in presynaptic terminals

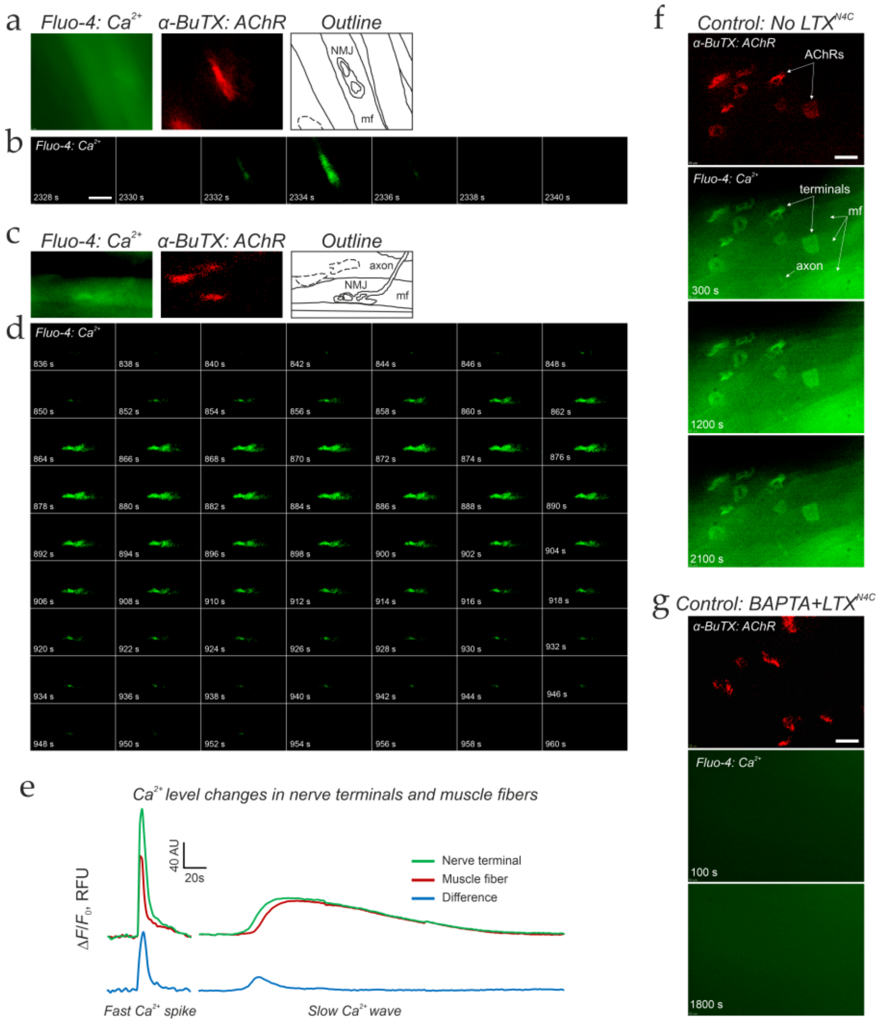

Figure S2. Nerve terminal-specific Ca^{2+}_{cyt} signals can be resolved even when mouse muscle fibers are unintentionally loaded with Fluo-4. In some preparations, Fluo-4-AM escaped the suction pipette and was taken up by both nerve terminals and their associated muscle fibers, necessitating validation of signal origin. (**a, b**) An NMJ exhibiting a Ca^{2+}_{cyt} spike. (**a**) Left: Fluo-4 fluorescence image showing dye in both nerve terminal and muscle fiber. Scale bar, 20 µm. Center: α-bungarotoxin Alexa Fluor 546 labeling of postsynaptic ACh receptors. Right: Schematic diagram. (**b**) Time-lapse images (identical contrast settings) showing a brief, intense Ca^{2+}_{cyt} spike localized to the nerve terminal. (**c, d**) A different NMJ exhibiting a Ca^{2+}_{cyt} wave. (**c**) Fluo-4 fluorescence image, ACh receptor labeling, and schematic. (**d**) Time-lapse images showing a slow wave of elevated Ca^{2+}_{cyt} flooding the nerve terminal. (**e**) Normalized Fluo-4 fluorescence traces ($\Delta F/F_0$) from the NMJs in (**a**) and (**c**). Green: nerve terminals; red: corresponding muscle fibers. Differential traces (blue) isolate the nerve terminal-specific signals, demonstrating that (i) the selected motor endings and muscle fibers form

functional motor units, and (ii) the observed presynaptic spikes and waves are free of contamination from muscle fiber Ca^{2+}_{cyt} changes. (**f–g**) Representative images from time-lapse recordings of Fluo-4-loaded nerve terminals. (**f**) Control (no stimulation). (**g**) Stimulation with LTX^{N4C} after pre-loading with BAPTA-AM. Scale bar, 50 μm.

3.3. LTX^{N4C} effects are mediated by LPHN1

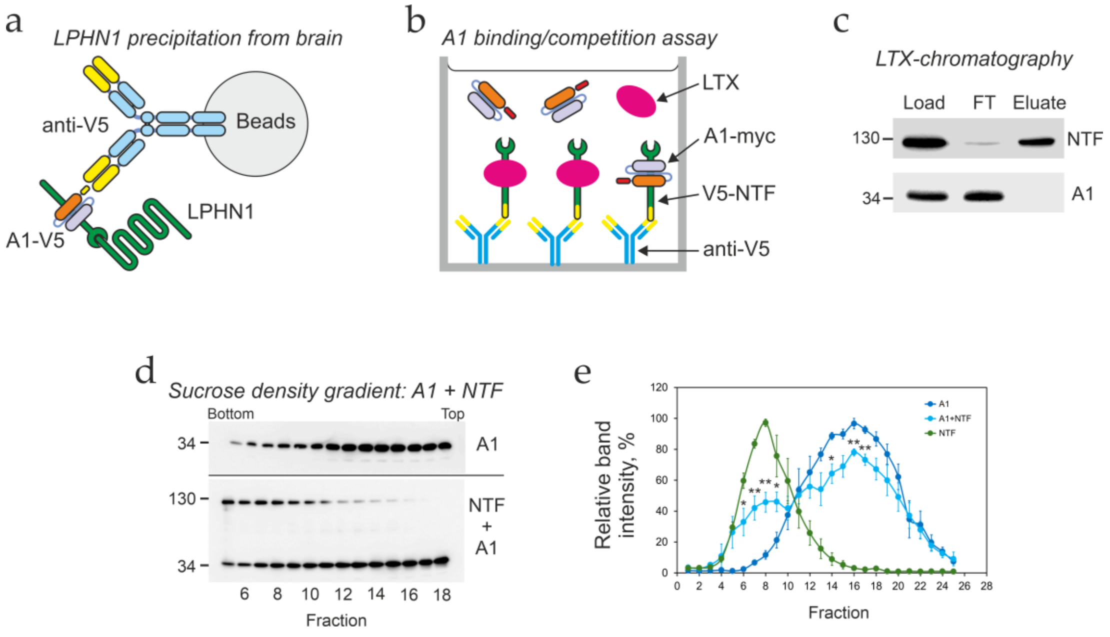

Figure S3. The scFv antibody A1 interacts with LPHN1 rather than αLTX. (**a**) Schematic of A1-myc-mediated precipitation of LPHN1 from solubilized mouse brain. (**b**) Diagram of two ELISA-based experiments for assaying (i) A1 affinity for LPHN1 (Figure 3f; αLTX was not added) and (ii) A1-αLTX competition for LPHN1 (Figure 3g; increasing concentrations of αLTX were applied). (**c**) A1 does not bind to αLTX. The toxin was immobilized on beads and used to pull down either a recombinant V5-tagged NTF of LPHN1 or A1-V5 from solution. Blots were probed with an anti-V5 antibody. FT, the flow-through fraction. (**d, e**) Assessment of the A1-LPHN1 interaction by sedimentation in sucrose density gradients. (**d**) Western blotting of fractions obtained by centrifugation of A1-V5 alone (upper blot) or together with the V5-tagged NTF of LPHN1 (lower blot) in 2-10% sucrose density gradients. Blots were probed with an anti-V5 mAb. Fraction numbers begin from the bottom of the gradient. (**e**) Quantification of A1 and NTF sedimentation. A1-V5 and V5-NTF bands on Western blots (as in **d**) were quantified, normalized to the area under the curve, and plotted against fraction number. The graph demonstrates that in the presence of NTF, a proportion of A1 shifts from its normal position in the gradient and co-sediments with NTF, indicating a specific interaction. Statistical significance of changes in A1 distribution is indicated above the A1-NTF curve: *, $p < 0.05$; **, $p < 0.01$; $n = 3$).

Continued

3.4. LPHN1 is presynaptic at the mouse and frog NMJ

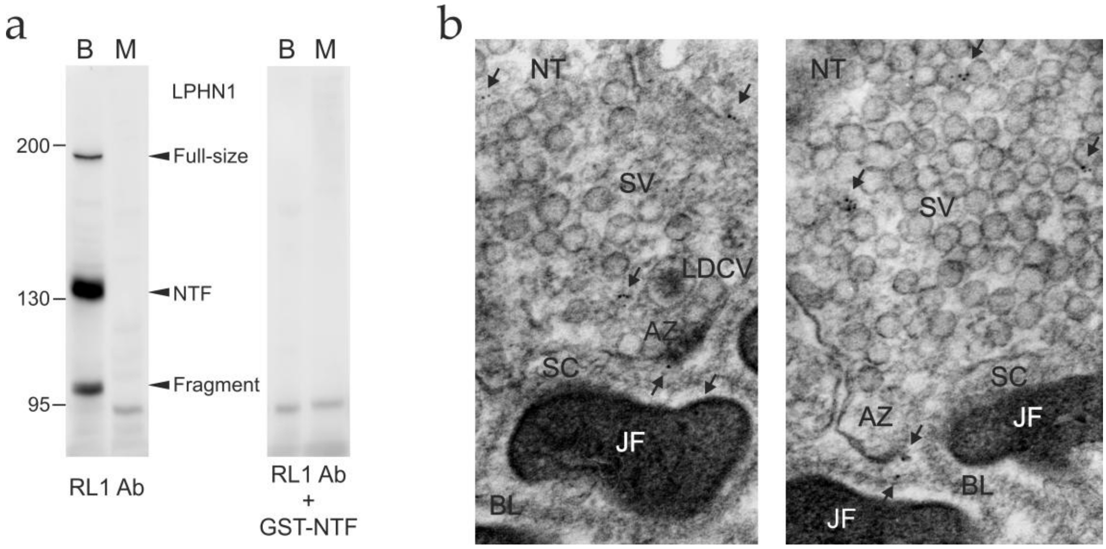

Figure S4. LPHN1 in the frog. (**a**) Specificity of the anti-LPHN1 antibody. Solubilized membranes from frog brain (B) and muscle (M) were analyzed by Western blotting using the affinity-purified anti-LPHN1 RL1 antibody. To confirm staining specificity, the antibody was pre-incubated with the GST-NTF fusion protein used for its affinity purification (RL1 + GST-NTF). This pre-absorption eliminated the LPHN1-specific signals, validating the antibody's specificity for LPHN1. (**b**) Immunogold electron microscopy of the frog NMJ with post-embedding labeling for LPHN1. The preparations were labeled with the affinity purified anti-LPHN1 antibody RL1 counterstained with 5-nm gold-conjugated goat anti-rabbit IgG antibody. Arrows indicate the positions of gold particles; AZ, active zone; BL, basal lamina; JF, junctional folds; LDCV, large dense-core vesicle; NT, nerve terminal; SC, synaptic cleft; SV, synaptic vesicles. Most LPHN1 labeling is found in the motor terminal, with only background staining of the muscle.

Continued

3.5. LPHN1 acts mainly via the Gαq/11 signaling pathway

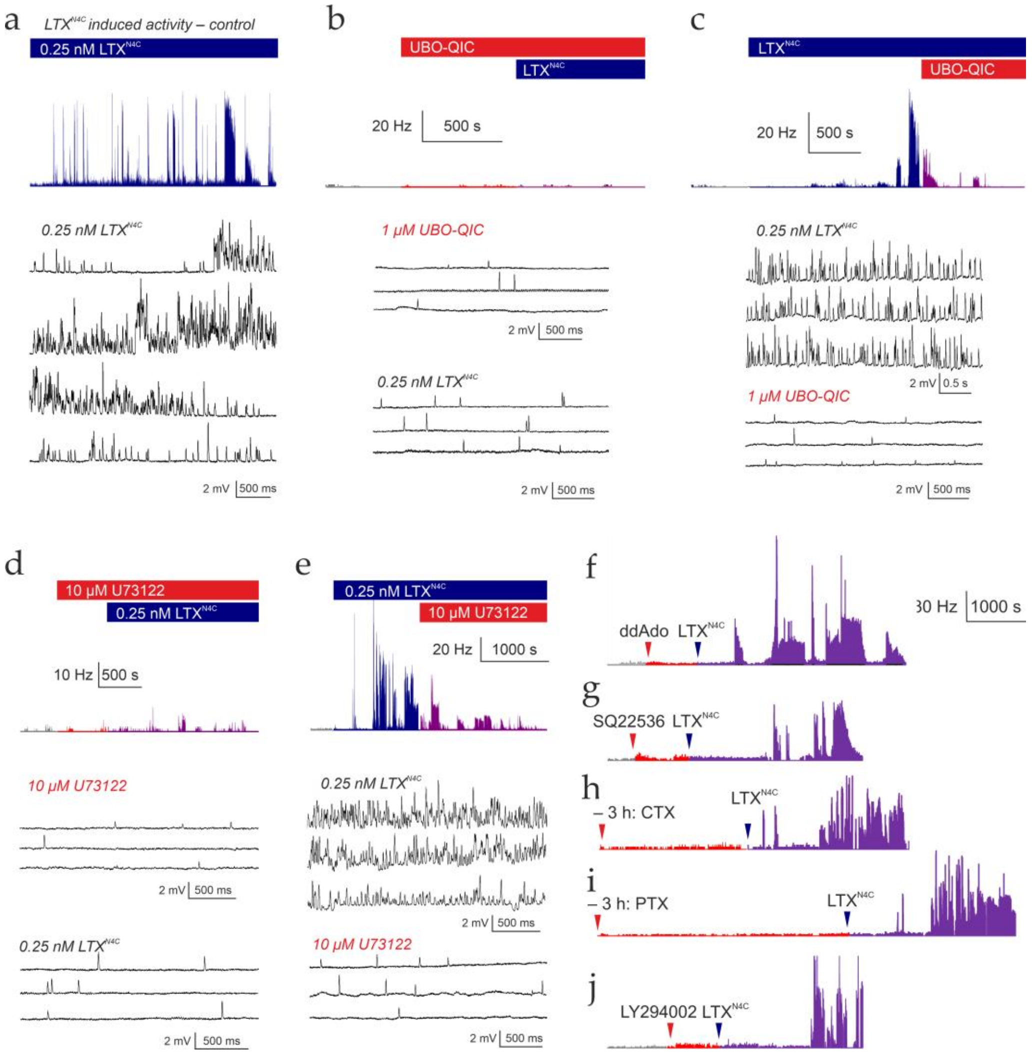

Figure S5. Gαq signaling is required for LTX^{N4C}-induced, LPHN1-mediated bursts of exocytosis. WT mouse nerve-muscle preparations were incubated in 2 mM $Ca^{2+}{}_e$ and stimulated with 0.25 nM LTX^{N4C} before or after treatment with the indicated inhibitors. (**a–e**) Top graphs summarize synaptic activity (MEPP frequencies in 1-s bins); bottom graphs show V_m recordings, with individual MEPPs or MEPP bursts as upward deflections. The bars above the top graphs indicate the time of addition and presence of the specified substances. (**a**) Control experiment with the addition of 0.25 nM LTX^{N4C} only. (**b, c**) The Gαq inhibitor UBO-QIC (1 μM) was applied before (**b**) or after (**c**) LTX^{N4C}. (**d, e**) The PLC inhibitor U73122 (10 μM) was applied before (**d**) or after (**e**) LTX^{N4C}. (**f–j**) Synaptic activity in 1-s bins from experiments where inhibitors were added before LTX^{N4C}: 100 μM ddAdo (**f**); 10 μM SQ22536 (**g**); 5 nM CTX (**h**); 20 nM PTX (**i**); 30 μM LY294002 (**j**). The times of addition of inhibitors and LTX^{N4C} are indicated by arrowheads.

Continued

3.6. Store-operated Ca²⁺ entry mediates the LPHN1-induced increase in spontaneous exocytosis

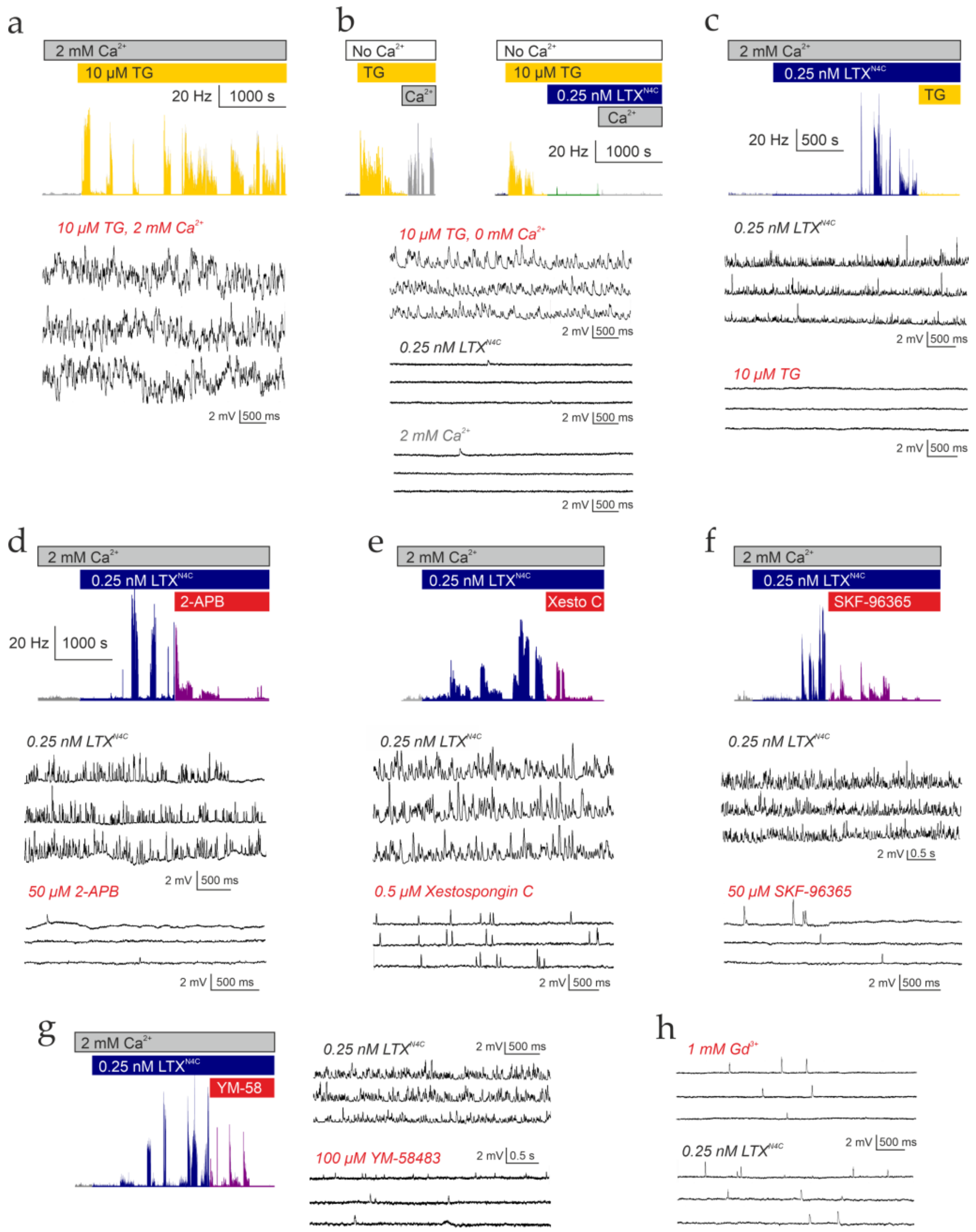

Figure S6. Ca^{2+} stores and SOCE are involved in the LTX-induced, LPHN1-mediated actions at the mouse NMJ. (**a–c**) Nerve-muscle preparations were stimulated with 10 μM TG in the presence or absence of 2 mM Ca^{2+}_e, alone or together with 0.25 nM LTX^{N4C}, as indicated. **(a–f)** Top graphs show synaptic activity in 1-s bins and the timing of substance addition. Bottom graphs show V_m recordings, with MEPPs appearing as upward deflections. (**a**) MEPP recordings in 2 mM Ca^{2+} and after TG addition. (**b**) MEPP recordings in 0 Ca^{2+}_e and after TG addition, followed by LTX^{N4C} and/or 2 mM Ca^{2+}. (**c**) MEPP recordings in 2 mM Ca^{2+} with TG added after the LTX^{N4C} effect had developed. (**d–f**) Preparations incubated in 2 mM Ca^{2+} were stimulated with LTX^{N4C}. After its effect developed, preparations were treated with: (**d**) 50 μM 2-APB; (**e**) 0.5 μM xestospongin C; (**f**) 50 μM SKF96365; or (**g**) 100 μM YM58483. (**h**) Preparations incubated in 2 mM Ca^{2+} and 1 mM Gd^{3+} were stimulated with 0.25 nM LTX^{N4C}.

Continued

3.7 The crucial role of VGCCs

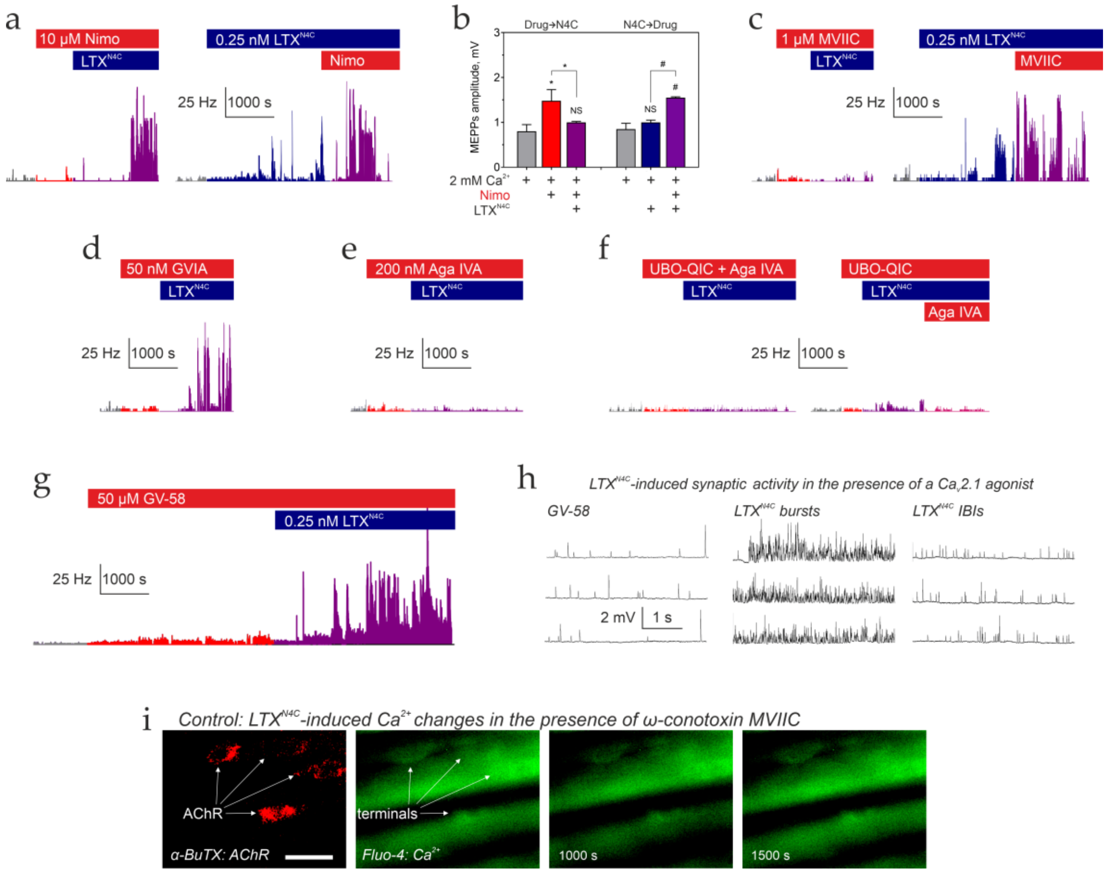

Figure S7. Cav2.1 channels provide a trigger for LTX^{N4C}-induced bursts of quantal release, while Cav1.x channels modify their dynamics. (**a**–**h**) Mouse muscle preparations were incubated in 2 mM Ca^{2+} and stimulated with 0.25 nM LTX^{N4C}. (**a**) Synaptic activity (in 1-s bins) induced by LTX^{N4C} added after or before 10 µM nimodipine, a Cav1.x channel blocker. Bursts are smaller but more frequent when nimodipine is added first and cease after ~15 min when nimodipine is added after LTX^{N4C}. (**b**) Nimodipine (10 µM) increases MEPP amplitude when added after or before LTX^{N4C}; n =6; N = 32. (**c**) Synaptic activity (in 1-s bins) induced by LTX^{N4C} added after or before 1 µM ω-conotoxin MVIIC. MVIIC inhibits LTX^{N4C} effects only when added first. (**d**–**g**) Synaptic activity (in 1-s bins) induced by LTX^{N4C} added after the following inhibitors or agonists (see text for details): (**d**) 50 nM ω-conotoxin GVIA; (**e**) 200 nM ω-agatoxin IVA; (**f**) 1 µM UBO-QIC and 200 nM ω-agatoxin IVA; (**g**) 50 µM GV-58. (**h**) V_m recordings in the presence of GV-58 alone or together with LTX^{N4C} (a representative burst and an IBI are shown). (**i**) LTX^{N4C} fails to induce characteristic Ca^{2+}_{cyt} spikes in the presence of a Cav2 blocker. Representative time-lapse Ca^{2+} fluorescence images of motor terminals preloaded with Fluo-4, incubated with 1 µM ω-conotoxin MVIIC and stimulated with 0.25 nM LTX^{N4C}.
